# Digital twinning of Cellular Capsule Technology: Emerging outcomes from the perspective of porous media mechanics

**DOI:** 10.1371/journal.pone.0254512

**Published:** 2021-07-12

**Authors:** Stéphane Urcun, Pierre-Yves Rohan, Wafa Skalli, Pierre Nassoy, Stéphane P. A. Bordas, Giuseppe Sciumè

**Affiliations:** 1 Institut de Biomécanique Humaine Georges Charpak, Arts et Metiers Institute of Technology, Paris, France; 2 Department of Engineering Sciences, Institute for Computational Engineering Sciences, Faculté des Sciences de la Technologie et de Médecine, Université du Luxembourg, Luxembourg, Luxembourg; 3 Institut de Mécanique et d’Ingénierie, Université de Bordeaux, Talence, France; 4 Institut d’Optique Graduate School, CNRS UMR 5298, Talence, France; University of Michigan, UNITED STATES

## Abstract

Spheroids encapsulated within alginate capsules are emerging as suitable *in vitro* tools to investigate the impact of mechanical forces on tumor growth since the internal tumor pressure can be retrieved from the deformation of the capsule. Here we focus on the particular case of Cellular Capsule Technology (CCT). We show in this contribution that a modeling approach accounting for the triphasic nature of the spheroid (extracellular matrix, tumor cells and interstitial fluid) offers a new perspective of analysis revealing that the pressure retrieved experimentally cannot be interpreted as a direct picture of the pressure sustained by the tumor cells and, as such, cannot therefore be used to quantify the critical pressure which induces stress-induced phenotype switch in tumor cells. The proposed multiphase reactive poro-mechanical model was cross-validated. Parameter sensitivity analyses on the digital twin revealed that the main parameters determining the encapsulated growth configuration are different from those driving growth in free condition, confirming that radically different phenomena are at play. Results reported in this contribution support the idea that multiphase reactive poro-mechanics is an exceptional theoretical framework to attain an in-depth understanding of CCT experiments, to confirm their hypotheses and to further improve their design.

## Introduction

As a tumor grows, it deforms the surrounding living tissues which, in turn, produce pressure on the growing tumor and causes strains and associated stresses. Mechano-biology focuses on these mechanical forces and their interplay with biological processes, which has been extensively studied experimentally [1–3]. Within this context, current mathematical models of tumor growth are becoming more and more reliable, complement experiments, and are useful tools for understanding, explaining and building upon these experimental findings [4–6].

This article focuses on the Cellular Capsule Technology (CCT), an experimental protocol developed by some of the authors in [7] where multi-cellular tumor spheroids (MCTS) were cultured within spherical porous alginate capsules. The latter, after confluence (*i.e*. when the MCTS comes in contact with the inner wall), work as mechanosensors i.e., from their deformation, one can retrieve the stress state within the MCTS. As envisioned in [7], the interaction pressure between the MCTS and the capsule, coming from the basic action-reaction principle, is a capital information since it could enable the prediction of stress-induced phenotype alterations to characterize cell invasiveness. To this aim, it is essential to quantify the critical pressure which induces the phenotype switch. Notably, it is also relevant to quantify the characteristic time of this process since one can infer that a relatively high pressure sustained by a cell during a relatively short time does not lead to phenotype modifications.

Using the measured interaction pressure as a direct discriminant to predict the occurrence of the phenotype switch is also very attractive due to the simplicity of the concept. However, as hypothesized in this paper, directly linking the interaction pressure and the phenotype switch could be a simplistic shortcut: behind the promising practical simplicity of the MCTS-capsule concept, there is a behavior which is not trivially explainable with basic physical concepts. Indeed, the interaction pressure is a quite overall consequence encompassing several mechanisms at a lower level of description. The mechanics of porous media, on which the digital twin of the CCT experiment proposed in this contribution is founded, has emerged as an excellent paradigm to model and possibly reveal these mechanisms offering a new perspective from which one can better interpret and exploit results of the CCT.

The internal structure of a tumor is typically highly heterogeneous. As a result, instead of analyzing the system with a homogeneous modeling framework—which is the only option to exploit patient-specific data and produce clinically-relevant tumor forecasts (e.g., see [8])—the MCTS is modeled in this contribution as a multiphase continuum consisting of tumor cells, interstitial fluid and an extracellular matrix. This is possible thanks to the richness of experimental data provided by CCT. Mathematical modeling enables retrieving of the stress of each phase from the Biot’s effective stress principle and the adopted multiphase formulation. The model is founded on the rigorous framework provided by the Thermodynamically Constrained Averaging Theory (TCAT) of [9].

To guarantee the scientific relevance of numerical results, the reliability of the model was evaluated using a crossing validation methodology. This allowed for a step-by-step customization of the mathematical model, obtaining a mechanistic formulation which remains predictive even when the experimental conditions of CCT experiment are modified. Systematic sensitivity analyses were helpful for the analysis and interpretation of results, allowing for a quantification of the relative relevance of mechanisms underlying tumor growth phenomenology.

The effective digital twinning of the MCTS-capsule system and emerging biophysical outcomes from the perspective of multiphase porous media mechanics constitute altogether the novelty of this work. Different existing modeling approaches can be phenomenological and too simplistic, or being of little use due to their complexity. The proposed mechanistic modeling approach contains the suitable degree of complexity to be representative as such a kind of experiment.

## Methods and model

CCT offers a framework to quantitatively assess the influence of mechanical stresses and its coupling with other biophysical factors impacting tumor cells proliferation and metabolism. Input data of the mathematical model can be retrieved from the CCT experimental conditions. Furthermore, numerical results in terms of pressure and displacement can be compared with those measured experimentally. This motivated the selection of CCT as reference experiments.

For the sake of clarity, the experimental observations reported by [7] together with some additional data provided by the authors are briefly summarized in the following subsection. The mathematical model and the *in silico* reproduction process are then presented.

### Encapsulated MCTS: Experimental procedure and observed phenomenology

MCTS cultures have been developed to overcome the limitations of 2D cultures which, inherently, lead to artifacts in cellular behaviors [10], and investigate biophysical aspects. They involve integrin-mediated adhesion, cell differentiation, or drug penetration as a readout for efficient delivery of active species, for which the 3D architecture of the tumor cell aggregate is suspected of having a significant contribution.

MCTS are generally formed and cultured in aqueous medium that we will refer to as “free conditions”. Recently, Alessandri et al. [7] developed a microfluidic technique, namely the CCT, to produce confined MCTS cultures. They demonstrated that confinement-induced mechanical forces inhibit the tumor growth as previously shown [1] but may also trigger a switch towards an invasive phenotype of the tumor cells, with a mechanism that differs from the one mediated by matrix rigidity-sensing [2]. The CCT allows for the encapsulation and growth of cells inside permeable, elastic, hollow hydrogel microspheres for the production of size-controlled MCTS. The hydrogel is made of calcium alginate which pore size of about 20 nm provides a permeability that permits free flow of nutrients and oxygen and ensures favorable conditions for cell proliferation.

**Fig 1:**
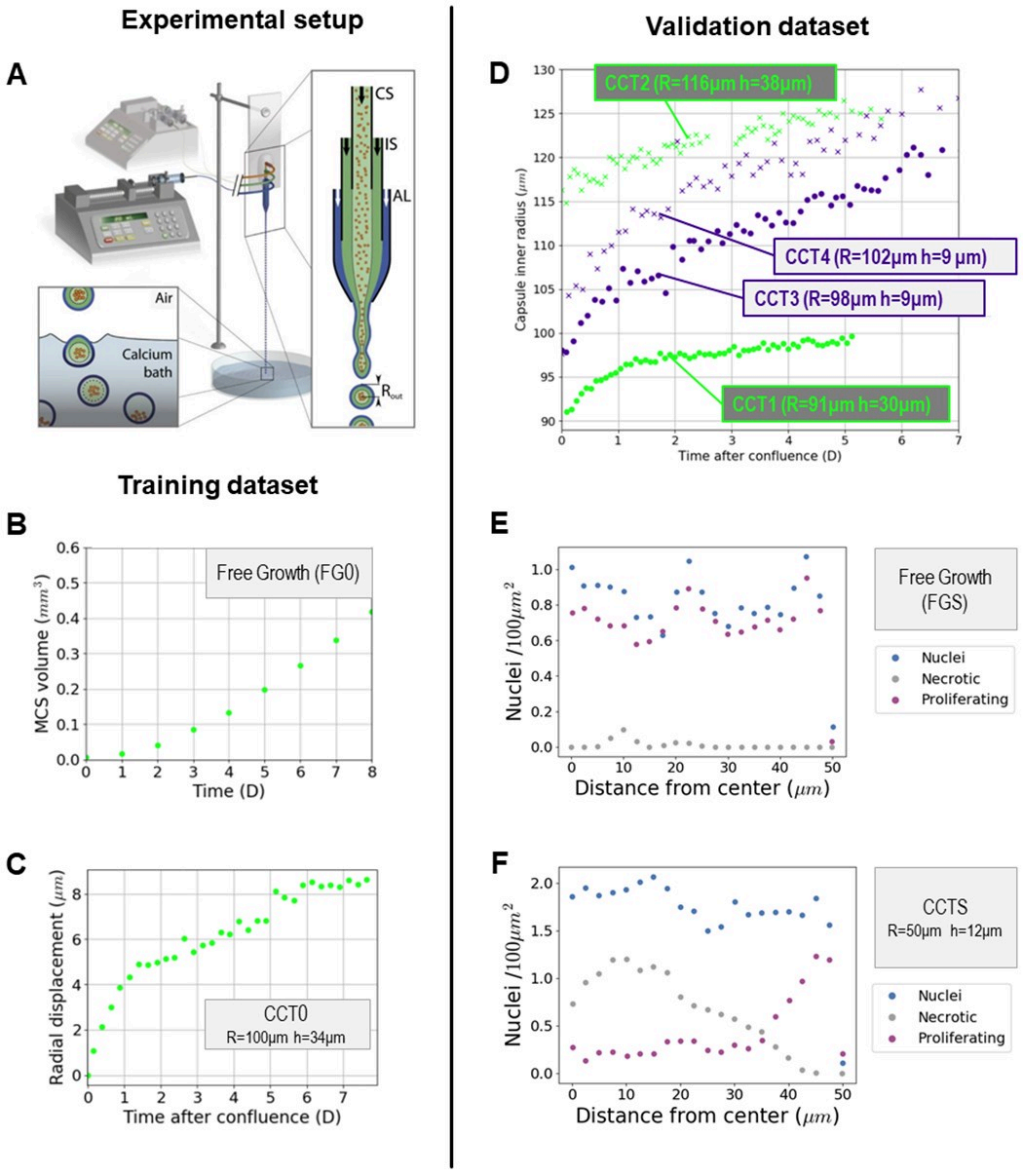
CCT experimental setup and data. **A** CCT microfluidic co-extrusion device; the enlarged view of the chip shows the three-way configuration, with cell suspension (CS), intermediate solution (IS), and alginate solution (AL), respectively, flowing into the coaligned capillaries. **B**, **C** Experimental training data: **B** free growth MCTS control group (FG0), the volume is monitored over a time span of 8 days; **C** encapsulated MCTS, the radial displacement of the capsule (CCT0) is monitored over a time span of 8 days. **D** Validation dataset: two capsules, denoted as thick CCT1 and CCT2; two capsules, denoted as thin CCT3 and CCT4. Their strains are monitored over a time span of 5 to 7 days. **E**, **F** Experimental quantification of cell nuclei (blue), proliferating cells (purple), and dead cells (gray) along the radius for small free (FGS), **E**, and small confined (CCTS), **F**, spheroids [7].

All experimental details and useful references are referenced in [7, 11]. Alginate spherical capsules are generated by co-extrusion using a 3D printed device composed of 3 co-axial channels (see Fig 1A): the outermost channel contains the alginate solution; the innermost capillary contains the cells in suspension; the intermediate channel contains an inert (sorbitol) solution that creates a barrier against calcium diffusion from the cell suspension to the alginate solution and subsequent plugging of the device. At flow rates in the 30–45 mL/h range for each channel, a composite liquid jet exits the nozzle and gets fragmented into composite droplets (of radius of the order of the diameter of the nozzle) due to the Rayleigh-Plateau instability. Composite droplets fall into a calcium bath. Since alginate crosslinks almost instantaneously in the presence of divalent ions, hydrogel shells encapsulating cells are readily formed. Then, cellular capsules are washed and transferred to a culture medium and placed in an incubator for temperature and atmosphere control. The capsule therefore serves as a micro-compartment for 3D cell culture.

In the present work, we used CT26 cell line which is derived from mouse colon carcinoma. Throughout the early stages, cells aggregate with each other. Upon proliferation, the MCTS first grows freely and increases the fraction of the capsule volume occupied by cells until the capsule is filled. By analogy with 2D cell monolayers in a Petri dish, this stage is named *confluence*. From then on, the MCTS interacts with the alginate shell and deforms it. Conversely, the dilated capsule exerts back a confinement pressure due to the action-reaction principle. In this respect, the post-confluent growing MCTS can be regarded as a tumor model that grows against the surrounding tissues and organs. While necrosis at the core of freely growing MCTS—due to nutrient diffusion limitation—is well known, similar necrosis can be observed in this experiment once the radius of the MCTS exceeds ∼ 250–300 *μ*m. This suggests that both confinement pressure and non-optimal oxygenation due to increased cell density cause these important measurable heterogeneities along the spheroid radius. In order to use the capsules as stress sensors, we need to characterize the material (Young’s modulus *E*_alg_) and morphological (shell thickness, inner and outer radii) properties. First, the dilatation of the alginate capsule was shown to exhibit an elastic deformation with negligible plasticity and no hysteresis. Young’s modulus was measured by atomic force microscopy indentation and osmotic swelling and has been found to be equal to *E*_alg_ = 68 ± 21kPa [7]. Secondly, and quite remarkably, i) the size of the capsules is set by the size of the nozzle, indicating that different capsule sizes can be obtained by fabricating another microfluidic chip; ii) the thickness of the shell can be tuned with a given chip by varying the ratio between the flow rates in the different channels. For instance, increasing the inner flow rate of the cell suspension with respect to the sum of all flow rates will make the shell thinner. On the basis of these measured parameters, capsules can truly serve as a biophysical dynamometer by using a relation that yields the variation of the inner pressure from the measurement of the capsule radial deformation.

To do so, we used time lapse phase-contrast video-microscopy, which is minimally invasive in terms of photo-toxicity and thus allows monitoring MCTS growth over several days (up to about a week). While MCTS are clearly visible because of the strong light absorption by living tissues, the alginate shell, which is made of 98% water, is fainter. Images were thus analyzed using a custom-made, gradient-based edge detection algorithm that allows tracking simultaneously the radius of the MCTS *R*_MCTS_ and the outer radius of the capsule *R*_out_. At time *t* = 0, the inner radius of the capsule *R*_in_ was measured manually and did not vary as long as confluence was not reached. In these pre-confluent stages, for any capsule thicknesses, the growth rate of the CT26 spheroid was not significantly different from the one derived from freely growing conditions, indicating that access to nutrients is not compromised by the presence of the alginate shell, whatever its thickness. When confluence was reached (i.e. when *R*_MCTS_ = *R*_in_), simultaneous monitoring of *R*_MCTS_ and *R*_out_ allowed computing the variation of the shell thickness, *b*, which was averaged over the capsule perimeter. After confluence, the behavior strongly deviated from that of the free MCTS case. Qualitatively, the same phenomenology was observed for all capsule thicknesses. However, capsule thickness appeared to be a significant determinant of MCTS confined growth when quantitative analysis was performed. Finally, we reported radial distributions of cell nuclei, dead cells and proliferative cells. These data were obtained i) by fixing the samples at different time points, typically before and after confluence, following standard protocols, ii) by embedding them in a resin and freezing for cryosection. Immunostaining for nucleus (DAPI) and cell proliferation (KI-67) was performed using two types of fluorophores. Confocal images were then analyzed using standard ImageJ routines for particle detection. To detect dead cells, we exploited the fact that nuclei of dead cells are smaller and brighter (when DAPI-stained) than the ones of living cells. We thus applied additional threshold criteria for brightness and size.

The pressure exerted by the MCTS was calculated using the formalism of thick-walled internally pressurized spherical vessels, and thus could be compared to the stress of each phase from the Biot’s effective stress principle of the multiphase system. Assuming that the alginate gel is isotropic and incompressible the radial displacement of the inner wall, *u*(*R*_in_), reads

$$\begin{array}{c} u\left(R_{\text{in}}\right)=\frac{3}{4}\frac{P_{\text{conf}}}{E}\frac{R_{\text{in}}}{1-{\left(R_{\text{in}}/R_{\text{out}}\right)}^{3}} \end{array}$$ (1)

where *P*_conf_ is the internal pressure, *E* is the Young’s modulus, and *R*_in_ and *R*_out_ are the inner and outer radii of the capsule, respectively. Alginate incompressibility also implies volume conservation of the shell. This gives the following constraint equation

$$\begin{array}{c} R_{\text{out}}^{3}\left(t\right)-R_{\text{in}}^{3}\left(t\right)=R_{\text{out}}^{3}\left(0\right)-R_{\text{in}}^{3}\left(0\right)=\delta \left(R_{0}^{3}\right) \end{array}$$ (2)

Using this equation, the two time variables *R*_in_(*t*) and *R*_out_(*t*) can be separated and pressure, *P*(*t*), written as a function of *R*_in_(*t*) only

$$\begin{array}{c} P_{\text{conf}}\left(t\right)=\frac{4}{3}E[1-\frac{1}{1+\delta \left(R_{0}^{3}\right)/R_{\text{in}}^{3}\left(t\right)}]\frac{u(R_{\text{in}}\left(t\right))}{R_{\text{in}}\left(t\right)} \end{array}$$ (3)

#### Experimental input data

First, we considered the denoted *training dataset*:

- for the free MCTS, denoted FG0, the volume was monitored over a time span of 8 days (Fig 1B);
- for the encapsulated MCTS, denoted CCT0, the radial displacement of the capsule (inner radius *R* = 100 *μ*m and thickness *h* = 34 *μ*m) was monitored for 8 days (Fig 1C).

The reliability of the mathematical model was then tested with a *validation dataset* (Fig 1D):

- Two thick capsules, denoted CCT1 (*R* = 91 *μ*m, *h* = 30 *μ*m) and CCT2 (*R* = 116 *μ*m, *h* = 38 *μ*m);
- Two thin capsules, denoted CCT3 (*R* = 98 *μ*m, *h* = 9 *μ*m) and CCT4 (*R* = 102 *μ*m, *h* = 9 *μ*m);

Sparse experimental data have been used to qualitatively measure the model emerging outcomes: the measurements of cell states (quiescent, proliferative, necrotic) of a *R* = 50 *μ*m free MCTS denoted FGS (Fig 1E) and a small thick capsule, denoted CCTS (*R* = 50 *μ*m, *b* = 12 *μ*m), 26 hours after confluence (Fig 1F). Concerning the stiffness of the alginate, *E*_alg_, a range *E*_alg_ = 68 ± 21kPa is considered in the simulation herein.

### The mathematical model: A physics-based description of the MCTS-capsule system

Our understanding of the physics and mathematical modeling in oncology has opened new perspectives thanks to our improved ability to measure physical quantities associated with the development and growth of cancer. Health research centers have been collaborating with engineers, mathematicians and physicists to introduce mechano-biology within clinical practice. In [12], the growth inhibition by mechanical stress has been used to reproduce patient specific prostate cancer evolution. This approach can be supplemented by biochemical and genetic approaches for the prediction of surgical volume for breast cancer [13] or the diffusion of chemical agent in pancreatic cancer [14]. For several years, robust clinically-oriented modeling frameworks have emerged (see the seminal article of [8]) wherein multi-parametric MRI patient sets have been processed to initiate patient specific modeling conditions [15]. Recent developments in [16] lead to a deep integration of mathematical oncology in the clinical process, from pre-clinical cell line growth used for model pre-calibration to multi-parametric MRI for patient specific calibration. The application of physics-based models is continuously growing with, for instance, *in vivo* modeling reproducing the vascular behavior and experimental validation using histological animal and human samples [5], the hybridization of poromechanics and cell population dynamics to mimic the effect of *in vivo* micro-environment [6] or a more classic pre-clinical *in vitro* tumor growth [17].

In physics-based modeling, three approaches are currently used to model cancer: discrete, continuum and hybrid (the reader is referred to more detailed descriptions in the works of [18]). Among continuum models, poromechanical ones (e.g., see [6, 17, 19, 20]) emerge today as valid approaches to model the interplay between biomechanical and biochemical phenomena. As extensively reported in the literature, appropriate elementary models for describing the response of tumor tissue to mechanical and environmental cues will depend on its timescale. At very short time scales (seconds to minutes), tumor cell response is dominated by the elastic response of the cytoskeleton giving tumors a solid-like behavior. When it lasts more than a second, the response of the cytoplasm to solicitations is essentially viscous and tumor tissue undergoes cellular reorganizations. This leads to large persistent deformations easily represented by a fluid-like viscoelastic model. In this contribution, tumor tissue is modeled neither as a fluid, nor as a solid, but as a multiphasic continuum consisting of a solid matrix (ECM) filled by Newtonian fluid phases.

In this paper the multiphase reactive poro-mechanical model of [20] is further developed and customized for digital twinning of CCT in order to reproduce numerically the experiment of [7], and gaining additional information not yet measurable *in vitro*.

**Fig 2:**
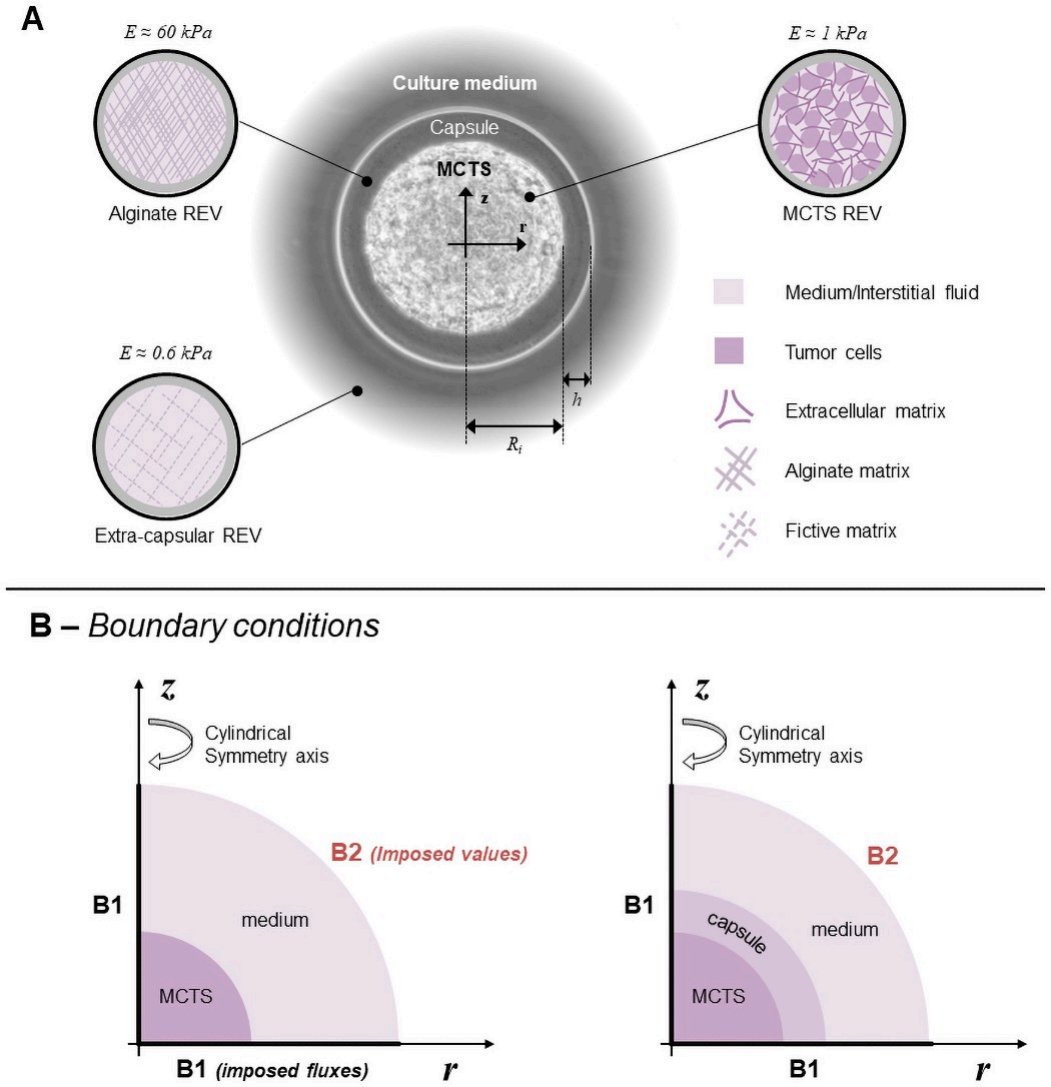
The capsule model. **A** Geometrical description of the capsule and assumed representative elementary volumes (REV): Three spatial domains modeled within the same mathematical framework: MCTS REV (consisting of tumor cells, interstitial fluid (IF) and extracellular matrix), the alginate shell (only IF phase within a solid scaffold of Young Modulus *E*_alg_ = 60kPa) and extra-capsular domain (only IF phase within a fictive solid scaffold of Young Modulus *E*_fict_ = 0.6kPa) enforced by the theoretical framework. **B** Computational boundary condition for the free (left) and confined (right) MCTS. At the bondary B1 symmetry conditions of no-normal flow/displacement are assumed while Dirichlet boundary conditions (e.g., prescribed oxygen concentration) are assumed at the boundary B2.

Our approach considers the tumor tissue as a reactive porous multiphase system: the extra-cellular matrix constitutes the solid scaffold while interstitial fluid (IF) and tumor cells (TC) are modeled as fluid phases. Hence, the mathematical model is governed by momentum and mass conservation equations of phases and species constituting the MCTS-capsule system. Once the capsule is formed, three different spatial domains can be defined (Fig 2A): the intra-capsular domain where is the tumor cells phase (*t*), the medium/interstitial fluid phase (*l*) and the extra cellular matrix phase (*s*) coexist; the alginate shell domain, where a solid scaffold phase (*s*) and the medium fluid phase (*l*) coexist; and the extra capsular domain where only the medium fluid phase (*l*) exists. In these three domains, strains are calculated according to the theory of poro-elasticity which always assumes the presence of a certain solid phase volume fraction constituting the porous/fibrous medium. Therefore, a certain proportion of the solid phase must always be present even in the extra-capsular domain. Despite this unrealistic condition enforced by the theoretical framework, the reliability of the model is only weakly affected, because the stiffness of this fictitious solid phase is two orders lower in magnitude than that of the alginate solid scaffold (Fig 2A). A unique physical model is defined for the three domains, with some penalty parameters (*e.g*., a low intrinsic permeability in the alginate domain) to avoid cell infiltration in the alginate shell. Oxygen advection-diffusion within the medium/interstitial fluid phase is considered. Oxygen acts as the limiting nutrient of TC with prolonged hypoxia leading to the cell necrosis.

The physical model consists of five governing equations:

- the solid scaffold *s* mass conservation
- the tumor cell phase *t* mass conservation
- the medium/liquid phase *l* mass conservation
- the advection-diffusion equation of oxygen in the medium/liquid phase *l*
- the momentum conservation equation of the multiphase system.

We have four primary variables: three are scalar and one vectorial.

- *p*^*l*^ the pressure of the medium/interstitial fluid
- *p*^*tl*^ the pressure difference between the cell phase *t* and the medium/interstitial fluid *l*
-
$\omega^{\bar{nl}}$ the mass fraction of oxygen
- **u**^*s*^ the displacement of the solid scaffold.

We also have two internal variables: the porosity *ε* and the TC necrotic mass fraction *ω*^*Nt*^. The evolution of porosity is calculated from the mass conservation equation of the solid phase while the mass fraction of necrotic cells is updated according to the tissue oxygenation in the TC phase (see [19]). We introduce two kinds of closure relationships for the system: mechanical and mechano-biological. Details about the derivation of the governing equation and these constitutive relationships are provided in the following sub-paragraphs.

#### The multiphase system

Three phases constitute the multiphase system, namely: the solid scaffold *s*, the medium/interstitial fluid *l* and the tumor cells phase *t*. Hence, at each point in the domain, the following constraint must be respected

$$\begin{array}{c} \varepsilon^{s}+\varepsilon^{t}+\varepsilon^{l}=1, \end{array}$$ (4)

where *ε*^*α*^ is the volume fraction of phase *α*. Defining the porosity *ε* as

$$\begin{array}{c} \varepsilon =1-\varepsilon^{s}, \end{array}$$ (5)

Eq 4 can also be expressed in terms of the saturation degree of fluid phase, *S*^*f*^ = *ε*^*f*^/*ε* (with *f* = *t*, *l*)

$$\begin{array}{c} S^{t}+S^{l}=1. \end{array}$$ (6)

#### Mass conservation equations

We express the mass conservation equations for each phase. We use a material description for the motion of the solid phase and a spatial description for the fluid phases, whose reference space is that occupied by the solid scaffold. As the solid is deformable, this reference space is not fixed in time but evolves according to the displacement of the solid phase. As a result, we express mass conservation equations for each phase and species in their material form with respect to the solid scaffold velocity. Mass conservation equations of solid, cell and interstitial fluid phases read:

$$\begin{array}{c} \frac{D^{s}}{Dt}(\rho^{s}\varepsilon^{s})+\rho^{s}\varepsilon^{s}\nabla \cdot v^{\bar{s}}=0, \end{array}$$ (7)

$$\begin{array}{c} \frac{D^{s}}{Dt}(\rho^{t}\varepsilon S^{t})+\nabla \cdot \left(\rho^{t}\varepsilon S^{t}v^{\bar{ts}}\right)+\rho^{t}\varepsilon S^{t}\nabla \cdot v^{\bar{s}}=\sum_{i\in l}\overset{i\to t}{\underset{,}{M}}\; \end{array}$$ (8)

$$\begin{array}{c} \frac{D^{s}}{Dt}(\rho^{l}\varepsilon S^{l})+\nabla \cdot \left(\rho^{l}\varepsilon S^{l}v^{\bar{ls}}\right)+\rho^{l}\varepsilon S^{l}\nabla \cdot v^{\bar{s}}=-\sum_{i\in l}\overset{i\to t}{\underset{,}{M}}\; \end{array}$$ (9)

where $\frac{D^{s}}{Dt}$ is the material time derivative with respect to the solid phase, *ρ*^*α*^ is the density of phase *α*, $v^{\bar{s}}$ is the velocity vector of the solid phase, $\sum_{i\in l}\overset{i\to t}{\underset{\;}{M}}$ is the total mass exchange (water, oxygen and other nutrients) from the interstitial fluid to the tumor due to cell growth and metabolism, $v^{\bar{ts}}$ is the relative velocity of cells and $v^{\bar{ls}}$ is relative velocity of the interstitial fluid with respect to the solid phase.

The tumor cell phase is a mixture of living (LTC) and necrotic tumor cells (NTC), with mass fraction $\omega^{\bar{Lt}}$ and $\omega^{\bar{Nt}}$, respectively. The following constraint applies

$$\begin{array}{c} \omega^{\bar{Lt}}+\omega^{\bar{Nt}}=1. \end{array}$$ (10)

Mass conservation equations for each fraction, assuming that there is no *intraphase* diffusion of both necrotic and living cells (whereas tumor cells phase has a diffusive term), read

$$\begin{array}{c} \begin{array}{c} \frac{D^{s}}{Dt}(\rho^{t}\omega^{\bar{Lt}}\varepsilon S^{t})+\nabla \cdot \left(\rho^{t}\omega^{\bar{Lt}}\varepsilon S^{t}v^{\bar{ts}}\right)+\rho^{t}\omega^{\bar{Lt}}\varepsilon S^{t}\nabla \cdot v^{\bar{s}}=\sum_{i\in l}\overset{i\to t}{\underset{\;}{M}}-\varepsilon^{t}r^{Nt}, \end{array} \end{array}$$ (11)

$$\begin{array}{c} \begin{array}{c} \frac{D^{s}}{Dt}(\rho^{t}\omega^{\bar{Nt}}\varepsilon S^{t})+\nabla \cdot \left(\rho^{t}\omega^{\bar{Nt}}\varepsilon S^{t}v^{\bar{ts}}\right)+\rho^{t}\omega^{\bar{Nt}}\varepsilon S^{t}\nabla \cdot v^{\bar{s}}=\varepsilon^{t}r^{Nt}, \end{array} \end{array}$$ (12)

where *ε*^*t*^ *r*^*Nt*^ is the death rate of tumor cells. Note that only one of Eqs 11 and 12 is independent: actually, one can be obtained subtracting the other from Eq 8 and accounting for the constraint Eq 10.

Oxygen is the only nutrient we consider explicitly. Another mass balance equation is introduced which governs the advection-diffusion of oxygen, *n*, within the interstitial fluid

$$\begin{array}{c} \begin{array}{c} \frac{D^{s}}{Dt}(\rho^{l}\omega^{\bar{nl}}\varepsilon S^{l})+\nabla \cdot \left(\rho^{l}\omega^{\bar{nl}}\varepsilon S^{l}v^{\bar{ls}}\right)+\nabla \cdot \left(\rho^{l}\omega^{\bar{nl}}\varepsilon S^{l}u^{\bar{nl}}\right)+\rho^{l}\omega^{\bar{nl}}\varepsilon S^{l}\nabla \cdot v^{\bar{s}}=-\overset{nl\to t}{\underset{,}{M}}\; \end{array} \end{array}$$ (13)

where $u^{\bar{nl}}$ is the diffusive velocity of oxygen in the interstitial fluid and $\overset{nl\to t}{\underset{\;}{M}}$ the oxygen consumed by tumor cells due to their metabolism and proliferation rate.

#### Momentum conservation equations

We neglect here the effect of gravitational body forces as their contribution is negligible compared to that of other forces. Furthermore, as we assume quasi-static processes and small difference in density between cells and aqueous solutions, inertial forces and the force due to mass exchange can also be neglected. These assumptions simplify the general form of the linear momentum balance equation given in [9] which becomes

$$\begin{array}{c} \nabla \cdot \left(\varepsilon^{\alpha }t^{\bar{\bar{\alpha }}}\right)+\sum_{K\in J_{c\alpha }}\overset{K\to \alpha }{\underset{\;}{T}}=0\;\left(\alpha =s,t,l\right), \end{array}$$ (14)

where $t^{\bar{\bar{\alpha }}}$ is the stress tensor of phase *α*, $J_{c\alpha }$ is the set phases connected to *α* and $\overset{K\to \alpha }{\underset{\;}{T}}$ is the interaction force between phase *α* and the adjacent phases. Summing Eq 14 over all phases leads to the momentum equation of the whole multiphase system as

$$\begin{array}{c} \nabla \cdot t^{\bar{\bar{T}}}=0, \end{array}$$ (15)

where $t^{\bar{\bar{T}}}$ is the total Cauchy stress tensor acting on the multiphase system.

Assuming that for relatively slow flow, the stress tensor for a fluid phase, *f*, can be properly approximated as

$$\begin{array}{c} t^{\bar{\bar{f}}}=-p^{f}1\;\left(f=t,l\right) \end{array}$$ (16)

where *p*^*f*^ is the averaged fluid pressure and **1** the unit tensor, Eq 14 which apply for a generic phase *α* (solid or fluid) can be expressed in an alternative form for fluid phases as [19]

$$\begin{array}{c} \varepsilon^{f}\nabla p^{f}+R^{f}\cdot \left(v^{\bar{f}}-v^{\bar{s}}\right)=0\;\left(f=t,l\right) \end{array}$$ (17)

where **R**^*f*^ is a symmetric second-order resistance tensor accounting for interaction between the fluid phase and the solid phase, *s*. Eq 17 can be rewritten as

$$\begin{array}{c} -K^{f}\cdot \nabla p^{f}=\varepsilon^{f}\left(v^{\bar{f}}-v^{\bar{s}}\right)\;\left(f=t,l\right), \end{array}$$ (18)

where **K**^*f*^ = (*ε*^*f*^)^2^(**R**^*f*^)^−1^ is called the hydraulic conductivity. The hydraulic conductivity depends on the dynamic viscosity of the flowing fluid, *μ*^*f*^, on the intrinsic permeability of the porous scaffold, *k*, and on the fluid saturation degree, *S*^*f*^, via a relative permeability function $k_{\text{rel}}^{f}\left(S^{f}\right)={\left(S^{f}\right)}^{A}$ (*A* depending on the fluid characteristics, see [21, 22]). As customary in biphasic flow problems, we set here $K^{f}=k\frac{k_{\text{rel}}^{f}\left(S^{f}\right)}{\mu^{f}}1$. Hence, the governing linear momentum conservation equation for tumor cells and interstitial fluid read respectively

$$\begin{array}{c} -k\frac{k_{\text{rel}}^{t}\left(S^{t}\right)}{\mu^{t}}\nabla p^{t}=\varepsilon^{t}\left(v^{\bar{t}}-v^{\bar{s}}\right), \end{array}$$ (19)

$$\begin{array}{c} -k\frac{k_{\text{rel}}^{l}\left(S^{l}\right)}{\mu^{l}}\nabla p^{l}=\varepsilon^{l}\left(v^{\bar{l}}-v^{\bar{s}}\right), \end{array}$$ (20)

#### Effective stress principle

We assume here that all phases are incompressible. However, the overall multiphase system is not incompressible, due to the presence of porosity that evolves according to the scaffold deformation. As all phases are incompressible, their densities *ρ*^*α*^ (with *α* = *s*, *t*, *l*) are constant and the Biot’s coefficient is equal to 1. Based on these premises, the total Cauchy stress tensor appearing in Eq 15 is related to the Biot’s effective stress as follows

$$\begin{array}{c} t^{\bar{\bar{E}}}=t^{\bar{\bar{T}}}+p^{s}1, \end{array}$$ (21)

where *p*^*s*^ = *S*^*t*^ *p*^*t*^ + *S*^*l*^ *p*^*l*^ is the so-called solid pressure, describing the interaction between the two fluids and the solid scaffold.

The chosen closure relationship for the effective stress $t^{\bar{\bar{E}}}$ is linear elastic:

$$\begin{array}{c} t^{\bar{\bar{E}}}=\bar{\bar{\bar{\bar{C}}}}:\epsilon \left(u^{\bar{s}}\right), \end{array}$$ (22)

with $\epsilon \left(u^{s}\right)=\frac{1}{2}\left(\nabla u^{\bar{s}}+{\left(\nabla u^{\bar{s}}\right)}^{T}\right)$ and $\bar{\bar{\bar{\bar{C}}}}\left(\lambda ,\mu \right)$ the fourth order elasticity tensor, reduced in Voigt notation:

$$\begin{array}{c} \bar{\bar{C}}\left(\lambda ,\mu \right)=(\begin{array}{cccccc} \lambda +2\mu & \lambda & \lambda & 0 & 0 & 0\\ \lambda & \lambda +2\mu & \lambda & 0 & 0 & 0\\ \lambda & \lambda & \lambda +2\mu & 0 & 0 & 0\\ 0 & 0 & 0 & \mu & 0 & 0\\ 0 & 0 & 0 & 0 & \mu & 0\\ 0 & 0 & 0 & 0 & 0 & \mu \end{array}) \end{array}$$ (23)

with the Lamé constants $\lambda =\frac{E\nu }{\left(1+\nu )(1-2\nu \right)}$ and $\mu =\frac{E}{2(1+\nu )}$.

*E* is the Young modulus of the solid scaffold and *ν* is its Poisson ratio.

#### Pressure-saturation relationship

**Fig 3:**
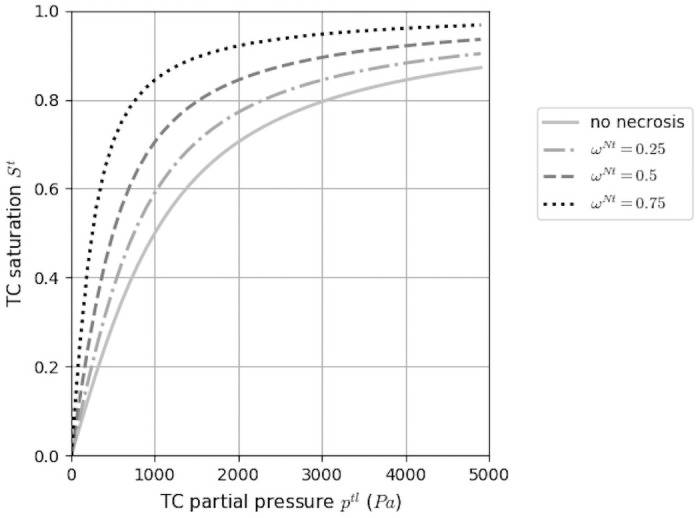
Constitutive relationship between tumor cell partial pressure and saturation. Tumor cell phase saturation *S*^*t*^, with the parameter *a* (fixed to 1kPa in the figure), evolving with the necrotic fraction of the phase $\omega^{\bar{Nt}}$.

The experimental measurement of cells density inside the capsule revealed a strong dependency to necrotic fraction $\omega^{\bar{Nt}}$. Hence, the pressure-saturation closure relationship has been improved with respect to that proposed in [20] to be more physically relevant and adapted to confinement situation

$$\begin{array}{c} S^{t}=\frac{2}{\pi }\text{arctan}(\frac{p^{tl}}{(1-\omega^{\bar{Nt}})a}), \end{array}$$ (24)

with *p*^*tl*^ pressure difference between tumor and interstitial fluid (i.e. *p*^*tl*^ = *p*^*t*^ − *p*^*l*^). The saturation is directly linked to the partial pressure of the phase and a constant parameter *a*, which accounts for the effect of cell surface tension and of the refinement of the porous network (see [22] for the biophysical justification of the proposed equation). Its influence is offset by the necrotic fraction of tumor cells, $\omega^{\bar{Nt}}$ (see Fig 3), which allows us modeling necrotic areas of very high cell density according to experimental evidence.

#### Nutrient diffusion

The tumor cells growth, metabolism and necrosis are regulated by a variety of nutrient species and intracellular signalling. However, without losing generality, in the present model one single nutrient is considered: oxygen. The case of multiple species can be easily obtained as a straightforward extension of the current formulation. The Fick’s law, adapted to a porous medium, was adopted to model diffusive flow of oxygen Eq 13:

$$\begin{array}{c} \omega^{\bar{nl}}u^{\bar{nl}}=-D^{nl}\nabla \omega^{nl} \end{array}$$ (25)

where *D*^*nl*^ the diffusion coefficient for oxygen in the interstitial fluid is defined by the constitutive equation from [21]

$$\begin{array}{c} D^{nl}=D_{0}^{nl}{\left(\varepsilon S^{l}\right)}^{\delta }, \end{array}$$ (26)

the exponent *δ* it set equal to 2 to account for the tortuosity of cell-cell interstitium where oxygen diffuse ([17, 20, 23]).

#### Tumor cells growth, metabolism and necrosis

Tumor cell growth is related to the exchange of nutrients between the IF and the living fraction of the tumor. The total mass exchange from IF to the tumor cell phase is defined as

$$\begin{array}{c} \sum_{i\in l}\overset{i\to t}{\underset{\;}{M}}=\gamma_{g}^{t}H\left(\omega^{\bar{nl}}\right)(1-H_{p}\left(p^{t}\right))(1-\omega^{\bar{Nt}})\varepsilon S^{t}, \end{array}$$ (27)

Note that $(1-\omega^{\bar{Nt}})\varepsilon S^{t}$ is the living fraction of the tumor. $\gamma_{g}^{t}$ is the tumor growth rate parameter, cell-line specific. H and $H_{p}$ are regularized step functions varying between 0 and 1, with two threshold parameters *σ*_1_, *σ*_2_, that is to say $H=H(\sigma ,\sigma_{1},\sigma_{2})$. When the variable *σ* is greater than *σ*_2_, H is equal to 1, it decreases progressively when the variable is between *σ*_1_ and *σ*_2_, and it is equal to zero when the variable is lower than *σ*_1_. H represents the growth dependency to oxygen:

$$\begin{array}{c} H\left(\omega^{\bar{nl}},\omega_{\text{crit}},\omega_{\text{env}}\right)=\{\begin{array}{c} 0\;\text{if}\;\omega^{\bar{nl}}\leq \omega_{\text{crit}}\\ \;\\ \frac{1}{2}-\frac{1}{2}\text{cos}\;\pi \frac{\omega^{\bar{nl}}-\omega_{\text{crit}}}{\omega_{\text{env}}-\omega_{\text{crit}}}\;\text{if}\;\omega_{\text{crit}}\leq \omega^{\bar{nl}}\leq \omega_{\text{env}}\\ \;\\ 1\;\text{if}\;\omega^{\bar{nl}}\geq \omega_{\text{env}} \end{array} \end{array}$$ (28)

**Fig 4:**
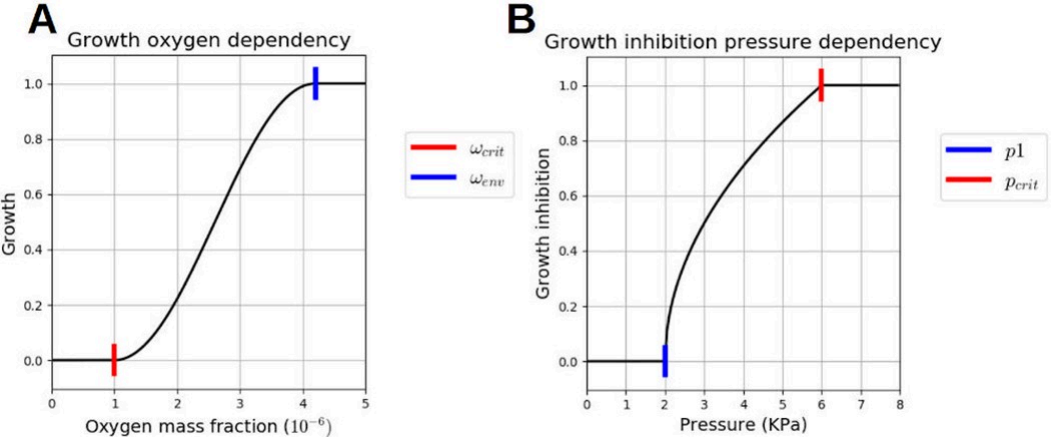
Two mechano-biological laws. **A** $H(\omega^{\bar{nl}},\omega_{\text{env}},\omega_{\text{crit}})$ . The TC growth and nutrient consumption are dependent to the oxygen mass fraction $\omega^{\bar{nl}}$. If it is lower than *ω*_crit_, the TC growth is stopped and the nutrient consumption is reduced to the metabolism needs only. If it is greater or equal to *ω*_env_, the growth and the nutrient consumption are maximum. **B** $H_{p}\left(p^{t},p_{\text{crit}},p_{1}\right)$. The TC growth and nutrient consumption are dependent to the TC pressure. If it is greater than *p*_1_, the 2 processes begin to be strongly affected and if the TC pressure reaches *p*_crit_, they are totally stopped.

*ω*_env_, the optimal oxygen mass fraction, is set to 4.2 * 10^−6^ which corresponds—according to Henry’s law—to 90mmHg, the usual oxygen mass fraction in arteries (see [24]). *ω*_crit_, the hypoxia threshold, is a cell line dependent parameter of tumor cells, which has been set to a very low value: 10^−6^ (≈ 20mmHg, for common human tissue cells, hypoxic level is defined between 10 and 20mmHg [25]) The function $H(\omega^{\bar{nl}},\omega_{\text{crit}},\omega_{\text{env}})$ is plotted in Fig 4A.

Function $\left(1-H_{p}\right)$ represents the dependency on pressure:

$$\begin{array}{c} H_{p}\left(p^{t},p_{1},p_{\text{crit}}\right)=\{\begin{array}{c} 0\;\text{if}\;p^{t}\leq p_{1}\\ \;\\ \sqrt{\frac{p^{t}-p_{1}}{p_{\text{crit}}-p_{1}}}\;\text{if}\;p_{1}\leq p^{t}\leq p_{\text{crit}}\\ \;\\ 1\;\text{if}\;p^{t}\geq p_{\text{crit}} \end{array} \end{array}$$ (29)

An example of the function $H_{p}\left(p^{t},p_{1},p_{\text{crit}}\right)$ is plotted in Fig 4B, we have set *p*_crit_ to 6 kPa as initial guess (in [1], they found a inhibitory pressure at 10 kPa) and *p*_1_, the pressure threshold when the inhibitory process starts, at 2 kPa.

As tumor grows, nutrients are taken up from the IF so that the sink term in Eq 13 takes the following form:

$$\begin{array}{c} \overset{nl\to t}{\underset{\;}{M}}=[\gamma_{g}^{nl}H\left(\omega^{\bar{nl}}\right)(1-H_{p}\left(p^{t}\right))+\gamma_{0}^{nl}\tilde{H}]\left(1-\omega^{\bar{Nt}}\right)\varepsilon S^{t}, \end{array}$$ (30)

Nutrient consumption from IF is due to two contributions: the growth of the tumor cells, as given by the first term within the square brackets in Eq 30, the metabolism of the living tumor cells, as presented in the second term. Thus, $\gamma_{g}^{nl}$ is related to the cell proliferation, as discussed above; whereas the coefficient $\gamma_{0}^{nl}$ relates to the cell metabolism. $\tilde{H}$ is an adaptation of the previous step functions for the cell metabolism:

$$\begin{array}{c} \tilde{H}\left(\omega^{\bar{nl}}\right)=\{\begin{array}{c} 1\;\text{if}\;\omega^{\bar{nl}}\geq \omega_{\text{env}}\\ \;\\ \frac{1}{2}-\frac{1}{2}\text{cos}\;\pi \frac{\omega^{\bar{nl}}}{\omega_{\text{env}}}\;\text{else} \end{array} \end{array}$$ (31)

The model does not discriminate between proliferating and quiescent cells, but the growth is subject to $H(\omega^{\bar{nl}},\omega_{\text{crit}},\omega_{\text{env}})$. To allow the comparison with the experimental proliferative cell quantities (see Fig 1E and 1F), the following relationship has been set:

$$\begin{array}{c} \omega_{\text{grow}}^{t}=\{\begin{array}{c} 0\;\text{if}\;\omega^{\bar{nl}}\leq \omega_{\text{crit}}\\ \;\\ S^{t}\frac{\omega^{\bar{nl}}}{\omega_{\text{env}}}\;\text{else} \end{array} \end{array}$$ (32)

$\tilde{H}$ is also used in the definition of hypoxic necrosis rate which reads

$$\begin{array}{c} \varepsilon S^{t}r^{Nt}=\gamma^{Nt}\left(1-\tilde{H}\left(\omega^{\bar{nl}}\right)\right)\left(1-\omega^{\bar{Nt}}\right)\varepsilon S^{t}, \end{array}$$ (33)

where *γ*^*Nt*^ = 0.01 is the necrotic growth rate. As the experimental data on necrosis were too sparse for this parameter identification (only a few stained-cell imaging), we have kept its generic value [26].

#### Initial parameter settings

As prescribed in [27], aiming for biological or clinical relevancy demands to investigate the choice of the initial values for each parameter. Some parameters are of physical nature (the IF dynamic viscosity, the oxygen mass fraction inside cell cultures), they can be, sometimes with great efforts, measured (or at least their values will be compared to the physical soundness). Others parameters belong more specifically to bio-poromechanical models in the mathematical oncology fields. Some of them have quite a theoretical nature (*e.g*., the ‘permeability’ of the ECM) while others have been experimentally measured at the cellular level (*e.g*., the oxygen consumption rate of EMT6/Ro cell line in [28]). Regarding these parameters, we have taken values used in previous numerical studies ([17, 20, 23, 29]) for MCTS cultured with other cell lines (human glioblastoma multiform and human malignant melanocytes), averaged these values, denoted ‘*generic*’, and used them as initial guess for identification of parameters of our CT26 cell line based MCTS.

When experimental data did not provide any relevant information on a parameter (*e.g*. for ECM stiffness and permeability), we chose to set them at their generic value. The following parameters have a non-negligible influence on the model outputs, and the closure relationships they belong to are explained in detail in the mathematical model section: $\gamma_{g}^{t}$ the TC growth rate (Eq 27), $\gamma_{g}^{nl}$ and $\gamma_{0}^{nl}$ the oxygen consumption rate due to growth and quiescent metabolism respectively (Eq 30), *a* the parameter tuning the joint impact of the ECM thinness and cell surface tension (Eq 24) and *μ*_*t*_ the TC dynamic viscosity, presented in Eq 19. Two other parameters, *p*_1_ and *p*_crit_, are introduced in this modeling. They represent thresholds which govern the inhibition of the proliferation (Eq 29) of cancer cells. The initial guess of *p*_crit_ has been chosen according to the work of [1, 2], and the initial guess of *p*_1_ has been set through the observation of the experimental data.

### In silico reproduction process

From the computational point of view, we aimed to a light and adaptable process: free, open source and compatible with any 2D or 3D geometry. For the model validation, we followed the convention of mathematical oncology proposed in [27]: two distinct sets of data for optimization and validation, the parameter set being fixed before validation. To measure the quality of the fits, we followed the method of [30]: the root mean square error (RMSE) relative to a reference, specified each time. The error on the numerical quantity *ξ*_num_ relative to a reference *ξ*_ex_, evaluated at *n* points is computed as:

$$\begin{array}{c} RMSE\left(\xi_{\text{num}},\xi_{\text{ex}},n\right)=\sqrt{\frac{1}{n}\sum_{k=1}^{n}(\frac{\xi_{\text{ex}}\left(k\right)-\xi_{\text{num}}\left(k\right)}{\xi_{\text{ex}}\left(k\right)}{)}^{2}} \end{array}$$ (34)

#### Computational framework

We implemented the above model in Python and C++ within the FEniCS framework [31], with an incremental monolithic resolution of the mixed finite element (FE) formulation. The simulations have been run with composite Taylor-Hood element $P_{3}\left(R^{2}\right)$, ${\left[P_{2}\left(R\right)\right]}^{3}$ (one vectorial and three scalar unknowns), a mesh element size of *dh* = 5 *μ*m and an implicit Euler scheme with *dt* = 1200s. All the details and analytical verification of the FE formulation are referenced in Appendix A. Computational framework. All the codes used in this article, analytical verification, integration along the inner radius of the capsule, free growth and confined growth, are available on Github, at https://github.com/StephaneUrcun/MCTS_mechanics

#### Initial and boundary conditions

The Fig 2 shows the two modeled configurations of MCTS (the free on the left and the confined on the right). Each mesh is half of a sphere as we also exploit symmetry with respect to a diametrical plane. For the three scalar variables, we prescribed Dirichlet boundary conditions along the outer radius of the domain *p*^*l*^ = 0, *p*^*tl*^ = 0 and $\omega^{\bar{nl}}=4.2\cdot 10^{-6}\;$ (according to Henry’s law, it corresponds to 90 mmHg, the usual oxygen mass fraction in arteries, see [24]) and no flux condition at *r* = 0 and *z* = 0. For the ECM displacement field **u**^*s*^, slip conditions $u_{r}^{s}{=0|}_{r=0}$ and $u_{z}^{s}{=0|}_{z=0}$ are used, and Dirichlet conditions **u**^*s*^ = **0** at the outer radius of the domain (see Fig 2).

#### Local sensitivity analysis

We performed a local sensitivity analysis to estimate Sobol sensitivity indices to assess the sensitivity of the FE solution to the input parameters. They have been performed on the FG0 and CCT0 training datasets, the freely growing and encapsulated MCTS. Further details of the process can be found in Appendix B. Sensitivity analysis.

First, we designed two cost functions, for FG0 and CCT0. The free growth cost function, *J*_free_, compares the experimental aggregate volume *V*_exp_ and the simulated volume from day one to day four.

$$\begin{array}{c} J_{\text{free}}=\sum_{i=1}^{4}(V_{\text{exp}}\left(D_{i}\right)-\int_{\Omega }\varepsilon_{i}S_{i}^{t}\;dx{)}^{2} \end{array}$$ (35)

The cost function for CCT0, *J*_conf_, compares two experimental quantities, the displacement of the internal radius **u**(*R*_in_) and the internal pressure calculated in Eq 3, with the corresponding model outputs **u**^*s*^ and *p*^*s*^, one day after confluence.

$$\begin{array}{c} \begin{array}{c} J_{\text{conf}}=\int_{\partial_{\text{Caps}}}<u\left(R_{\text{in}}\right)-u^{s}>\;ds+\int_{\partial_{\text{Caps}}}{\left(P_{\text{conf}}-p^{s}\right)}^{2}\;ds \end{array} \end{array}$$ (36)

where ∂_Capsule_ stands for “along the inner radius of the capsule”.

The results of the two configurations, with the parameters at their initial values used for the sensitivity analysis can be found in Appendix B. Sensitivity analysis, S5 Fig.

Secondly, the 7 parameters (*μ*_*t*_, *a*, $\gamma_{g}^{t}$, $\gamma_{g}^{nl}$, $\gamma_{0}^{nl}$, *p*_1_, *p*_crit_) were disturbed one at a time respectively to a [−10, −5, −2, −1, +1, +2, +5, +10]% grid. The variations of *J*_free_ and *J*_conf_ were interpolated by a linear model, which enables the calculation of first-order Sobol indices. The influence of the *i*^*th*^ parameter was deduced from the slope *θ*_*i*_ of the linear fit. The Sobol index *S*_*i*_ was calculated as follows:

$$\begin{array}{c} S_{i}=\frac{\theta_{i}^{2}}{\sum_{i}\theta_{i}^{2}} \end{array}$$ (37)

Finally, the 21 parameters tuples were evaluated at the 2 extreme values of the grid, [−10, +10]%, for each configuration. The variations of *J*_free_ and *J*_conf_ were interpolated by a second-order polynomial model. This leads to the computing two types of Sobol indices: *S*_*i*_ for the influence of the parameter *i* and *S*_*ij*_ for the influence of each couple (*i*, *j*) of parameters.

$$\begin{array}{c} S_{i}=\frac{\theta_{i}^{2}}{\sum_{i}\theta_{i}^{2}+\sum_{ij,i>j}\theta_{ij}^{2}}\;\text{and}\;S_{ij}=\frac{\theta_{ij}^{2}}{\sum_{i}\theta_{i}^{2}+\sum_{ij,i>j}\theta_{ij}^{2}} \end{array}$$ (38)

#### Parameter identification and model validation

For both configurations, the identification of relevant parameters was based on sensitivity profiles, as we identified the set of parameters that gathered at least 90% of the variance of the solution. Then, the selected parameters were identified by a Nelder-Mead simplex algorithm (in the Python library SciPy, method minimize, option Nelder-Mead). On the one hand, this algorithm has the advantage of not requiring the computation of the system gradient to the parameters. On the other hand, it generally converges to a local minimum. To avoid this phenomenon, a large range of initial guess was tested: [−20, +20]% around the *generic* values for $\mu_{t},\;a,\;\gamma_{g}^{t},\;\gamma_{g}^{nl}$ and $\gamma_{0}^{nl}$ and [−50, +50]% around the initial guess of *p*_1_ and *p*_crit_, as we have not previous literature values. All the parameters having a physical meaning, their values were bound to the physical and physiological values reported in the literature, and we choose not to extend this range. In the CCT0 configuration, considered as the representative case by the team of [7], the parameters of the MCTS cell line were identified with the mean experimental value of the alginate stiffness: *E*_alg_ = 68kPa; it is important to note that this study is not aiming to identify the stiffness of this biomaterial.

**Fig 5:**
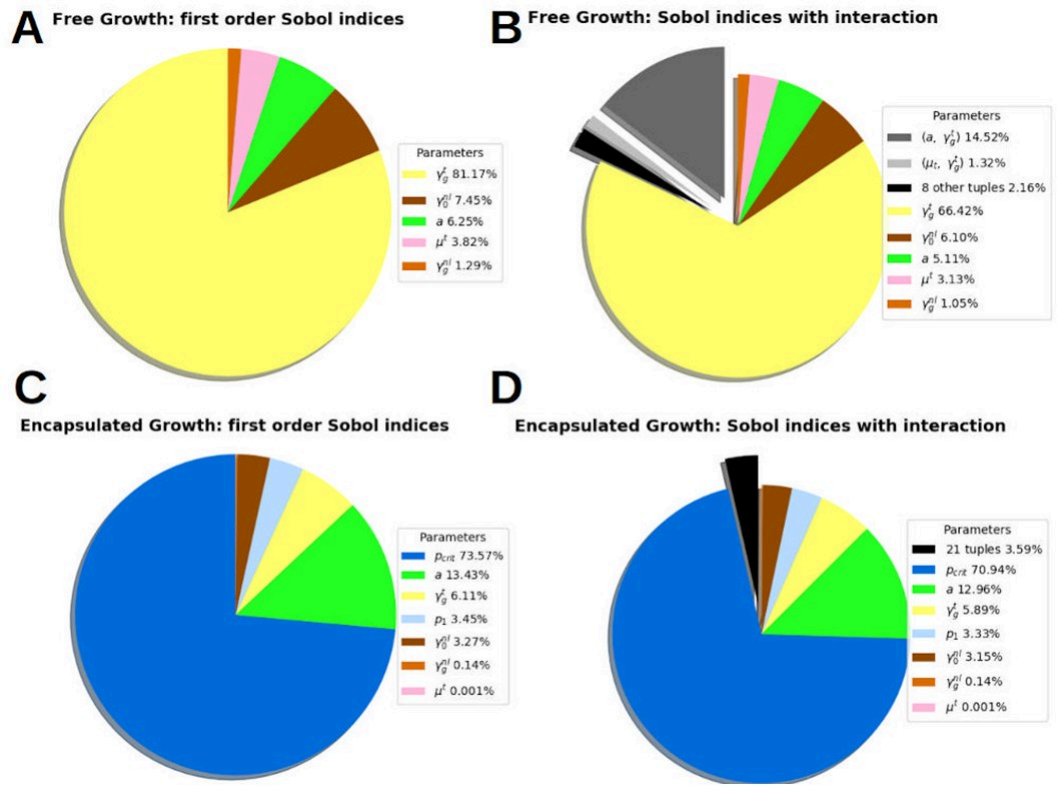
Validation of the calibrated parameters. Alginate Young’s modulus was estimated in [7] as *E*_alg_ = 68 ± 21kPa. Simulations with the extreme values of *E*_alg_ give the range of possibilities of the optimized set predicted with the model, denoted *Model range due alginate stiffness* in the plots. Experimental results, green dotted; Numerical results with the optimized parameter set, black; Modeling range (*E*_alg_ = 68 ± 21kPa), grey filled. **A**. Left, free MCTS control group, Time (Day) versus MCTS volume (mm^3^), the model fits the experimental data with a *RMSE* = 0.031. Right, CCT0. The fit uses the mean experimental value of the alginate stiffness *E*_alg_ = 68 kPa. The model fits the experimental data with a *RMSE* = 0.124. **B** Validation of the identified parameters on 2 thick capsules, CCT1 and CCT2. Time (Day) versus Capsule radius (*μ*m). The experimental points are in the modeling range. Both capsule fit with *E*_alg_ = 52.5 kPa and *E*_alg_ = 70 kPa respectively. **C** Validation of the identified parameters on 2 thin capsules, CCT3 and CCT4. Time (Day) versus Capsule radius (*μ*m). Left, one capsule is fitted with *E*_alg_ = 54kPa; right, the proposed fit is given with *E*_alg_ = 47kPa. However, an important part the experimental points are outside of the modeling range.

To evaluate the reliability of the identified parameters, an author of this article and member of the team of [7] have jointly provided unpublished experimental results of encapsulated MCTS, CCT1 to 4 and CCTS, namely the validation dataset. As their alginate stiffness is not known (the Young’s modulus of the alginate is estimated to be *E*_alg_ = 68 ± 21kPa), two simulations were run for each capsule with the extreme values of *E*_alg_. This provided the range of modeling possibilities of the identified parameters (Fig 5, grey range). For each capsule, we tested a set of *E*_alg_ values and proposed an indicative fit with the value of *E*_alg_ with the lower RMSE.

## Results

Based on a detailed sensitivity study, the identified set of parameters was tested and cross-validated on unpublished experimental results (Fig 1C–1E) provided by the same team of [7]. Numerical simulation also provides a wide output of qualitative results which are presented and interpreted. At the end of the section, we show that the model outputs allow predicting, with a reasonably good accuracy, experimental TC saturation and its necrotic fraction, although these quantities were not exploited for the model calibration.

### Sensitivity analysis

**Fig 6:**
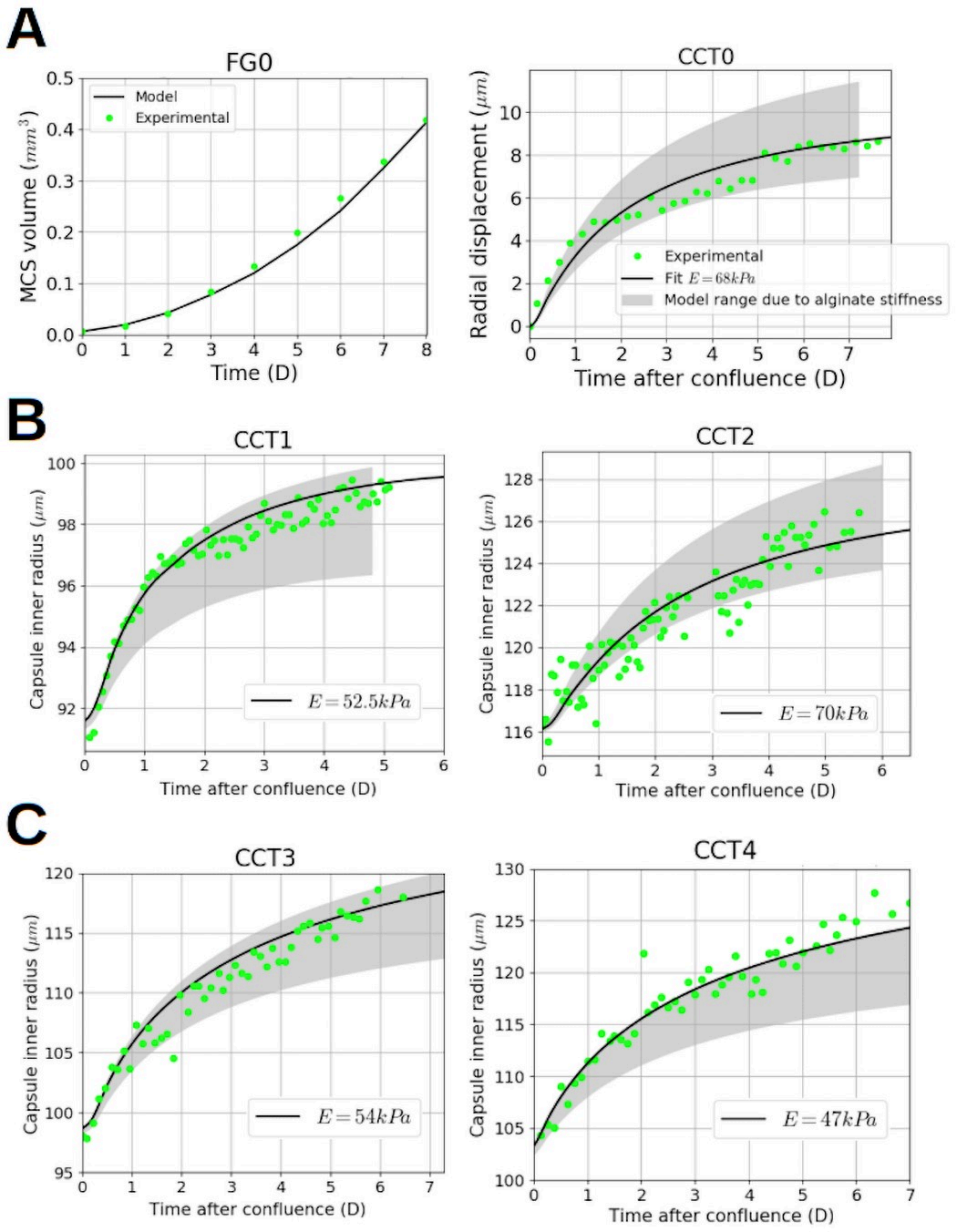
Sobol indices of the solution sensitivity. Sobol indices of the solution sensitivity on 7 parameters: *μ*_*t*_ the TC dynamic viscosity, the parameter *a* accounting for the joint impact of the ECM thinness and cell surface tension, $\gamma_{g}^{t}$ the TC growth rate, $\gamma_{g}^{nl}$ and $\gamma_{0}^{nl}$ the oxygen consumption rate due to growth and quiescent metabolism respectively, *p*_1_ and *p*_crit_, two thresholds which govern the pressure-induced inhibition of the TC proliferation. Free MCTS configuration (top row). **A First-order analysis**: Only 5 parameters remain, the governing parameter is $\gamma_{g}^{t}$, the tumor cells growth rate, the sensitivity of the solution on the pressure parameters, *p*_1_ and *p*_crit_, is 0. **B Interaction**: among 10 parameters tuples, one is significant $\left(a,\gamma_{g}^{t}\right)$ accounting for 14.5% of the solution variance. Thus, these two parameters are not independent and should identified together. The total variance of the solution shows that, considering all the interactions, the influence of each parameter alone is not qualitatively changed: $\gamma_{g}^{t}$ from 81.1% to 66.4%, $\gamma_{0}^{nl}$ from 7.4% to 6.1%, *a* from 6.2% to 5.1%. Encapsulated MCTS configuration (bottom row). **C First-order analysis**: the governing parameter is *p*_crit_ the inhibitory pressure of tumor cells growth (73.5% of the solution variance). **D Interaction**: the sum of 21 parameters tuples represents 3.6% of the solution variance (the detail of 21 tuples can be found in Appendix B. Sensitivity analysis, S5 Table).

Fig 6 shows the results of first-order and second-order interaction analyses, for the free and encapsulated configurations respectively. Clearly distinct profiles were obtained.

In the free growth control group FG0, the governing parameter is $\gamma_{g}^{t}$ the tumor cells growth rate (first-order index, $S_{\gamma_{g}^{t}}=81\%$, with interactions $S_{\gamma_{g}^{t}}=66.4\%$). In decreasing order of magnitude, we observed that two parameters are not negligible: $\gamma_{0}^{nl}$ the oxygen consumption due to metabolism (first-order index 7.45%, with interactions 6.10%) and *a*, the parameter determining the joint impact of the ECM thinness and cell surface tension (first-order index 6.25%, with interactions 5.11%). The important difference between the 2 Sobol indices of $\gamma_{g}^{t}$ is explained by the only non-negligible interaction between two parameters: $\gamma_{g}^{t}$ and *a* ($S_{\left(a,\gamma_{g}^{t}\right)}=14.5\%$, see Fig 6, right). This important interaction is indicative of the significant roles that ECM properties and cell-cell adhesion exert on proliferative-migration behavior (this is widely described in literature, see for instance [2]). Our modeling approach can reproduce mechanistically how these properties impact the overall observed phenomenology of tumor growth. These two parameters, $\gamma_{g}^{t}$ and *a*, are not independent and should be identified together.

For all parameter perturbations in the first-order and second-order interaction analyses, the pressure of the TC phase *p*^*t*^ = *p*^*l*^ + *p*^*tl*^ was less than 1KPa. Thus, the first threshold of growth inhibitory due to pressure *p*_1_ was never reached and, *a fortiori*, the critical threshold of total inhibition *p*_crit_. Thus, the sensitivity of the FE solution to *p*_1_ and *p*_crit_ was 0. The 3 parameters $\gamma_{g}^{t}$, *a* and $\gamma_{0}^{nl}$ has therefore been optimized for the free configuration.

For the encapsulated configuration CCT0, the governing parameter is the critical inhibitory pressure *p*_crit_ (first-order $S_{p_{\text{crit}}}=73.5\%$, with interaction 70.9%). $\gamma_{g}^{t}$, *a* and $\gamma_{0}^{nl}$ has already been identified for the free configuration, the only non-negligible parameter remaining is *p*_1_ (first-order index $S_{p_{1}}=3.4\%$, with interaction 3.3%). The difference between Sobol indices of first-order and interactions is weak. Indeed, the 21 parameters tuples capture only 3.6% of the solution variance. Thus, in the encapsulated configuration the parameters can be considered non-correlated and be identified separately.

These results, in the encapsulated configuration CCT0, show that the mechanical constraint is the phenomenon that determines the overall growth phenomenology provided by the mathematical model.

### Calibration

The three governing parameters $\gamma_{g}^{t},\gamma_{0}^{nl},a$ for the free MCTS configuration were identified using the Nelder-Mead simplex algorithm and fitted to the experimental data with a *RMSE* = 0.031. To be physically relevant, the same parameter set should be shared by the two configurations. These three parameters being calibrated, we consider they describe a subset of the mechano-biological states: growth without mechanical constrain. The parameters being independent in the encapsulated configuration, the identified values of these parameters are thereafter fixed in this configuration. Its two remaining parameters *p*_1_, *p*_crit_ (74% of the variance) were identified using the same algorithm. We fitted the experimental data of the encapsulation with a *RMSE* = 0.124. Fig 5A shows the two configurations fitted with the following set of parameters: $\gamma_{g}^{t}=3.33\cdot 10^{-2}\text{kg}/\left({\text{m}}^{3}.\text{s}\right),\;\gamma_{0}^{nl}=6.65\cdot 10^{-4}\text{kg}/\left({\text{m}}^{3}.\text{s}\right),\;a=890\;\text{Pa},\;p_{1}=1432\;\text{Pa},\;p_{\text{crit}}=5944\;\text{Pa}$ (see Table 1). This set is cell line specific, only relevant for CT26 mouse colon carcinoma.

**Table 1:** Parameters for the CT26 cell line. Source of the generic values: [17, 20, 23, 29].

Parameter	Symb.	Generic	Unit	Optimized
ECM network thinness	*a*	800	Pa	890
Dynamic viscosity of TC	*μ*_*t*_	36	Pa.s	negligible
TC growth rate	$\gamma_{g}^{t}$	4 ⋅ 10^−2^	kg/(m^3^.s)	3.33 ⋅ 10^−2^
TC growth *O*_2_ consump.	$\gamma_{g}^{nl}$	4 ⋅ 10^−4^	kg/(m^3^.s)	negligible
TC metabolism *O*_2_ consump.	$\gamma_{0}^{nl}$	6 ⋅ 10^−4^	kg/(m^3^.s)	6.65 ⋅ 10^−4^
Start TC growth inhibitory	*p*_1_	1800	Pa	1432
Stop TC growth	*p*_crit_	4000	Pa	5944

### Validation

Unpublished experimental results of encapsulated MCTS, both thick and thin, were used as validation dataset (Fig 1D). Each capsule had its own radius *R* and thickness *h* and two simulations were run with the extreme experimental values of the alginate stiffness (*E*_alg_ = 47 kPa and *E*_alg_ = 89 kPa).

Fig 5A right shows the range of modeling possibilities of the identified parameters on the training data CCT0, respectively to the alginate stiffness range. The parameter set was identified with the mean stiffness value (*E*_alg_ = 68 kPa).

Fig 5B shows that the modeling range on two thick capsules, CCT1 and CCT2, is in accordance with the experimental results. Two fits with an alginate stiffness at *E*_alg_ = 52.5 kPa and *E*_alg_ = 70 kPa respectively are proposed.

Fig 5C shows results relative to two thin capsules, CCT3 and CCT4. The dynamic is properly reproduced by the model for both capsules which are importantly deformed (the strain is of 16% for the left one and 20% for the right one). In the CCT4 case, the model shows some limitations, the proposed fit being at the minimum of its experimental stiffness value *E*_alg_ = 47 kPa. Nevertheless, the presented cross-validation demonstrates that this mechanistic mathematical model can adapt to different geometries and thickness without losing its relevance. Focusing on the left graphs in Fig 5B and 5C we can note that, with the same parameter set and almost the same alginate stiffness (B Left, *E*_alg_ = 52.5 kPa and C Left, *E*_alg_ = 54 kPa), the model reproduces an experimental strain of 8% and 16% respectively. The difference between the two strains is induced by the geometrical effect due to the capsule thickness, which impacts on the evolution of internal stresses, cell growth and oxygen consumption.

### Qualitative results and emerging outcomes

**Fig 7:**
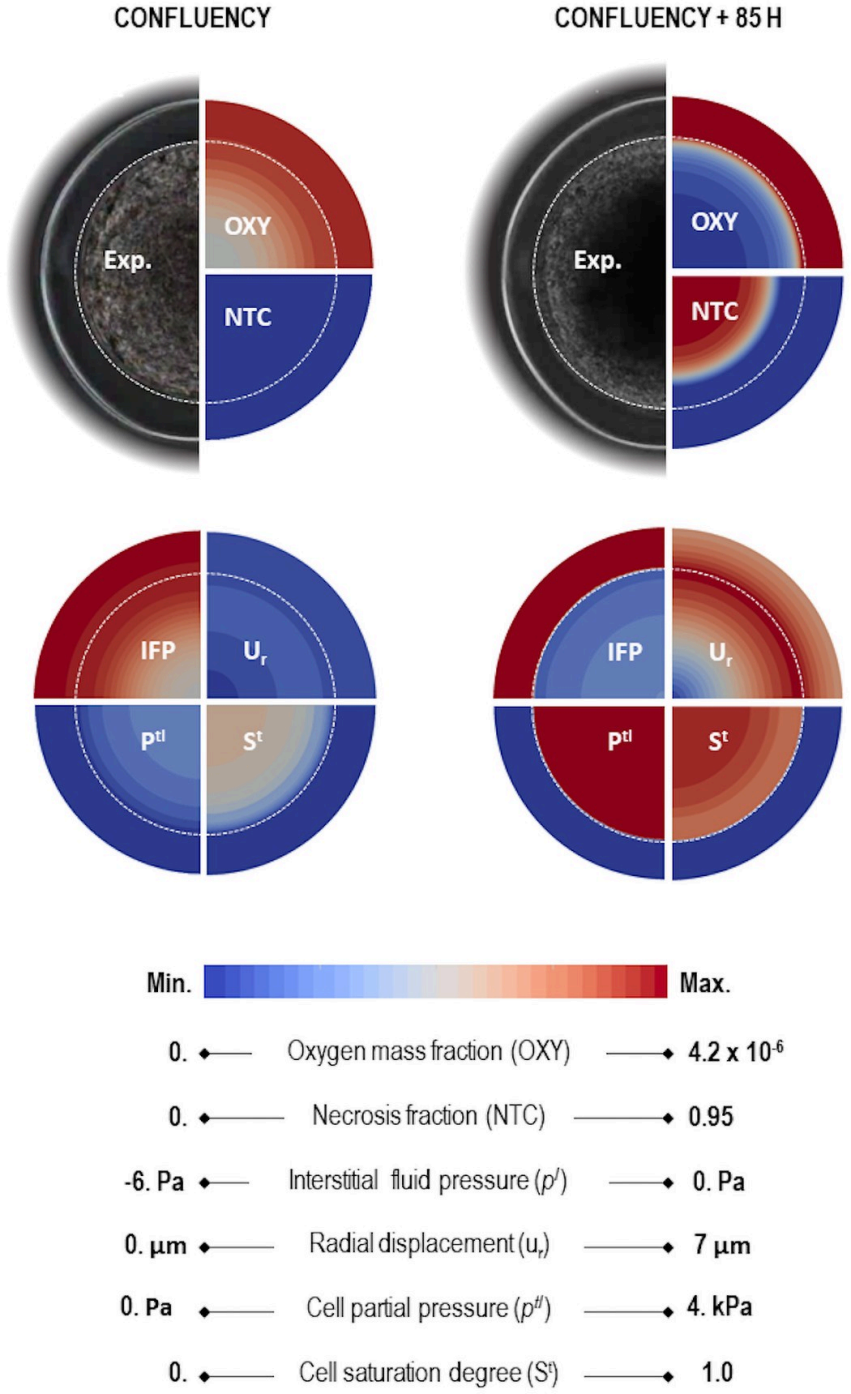
Experimental microscopy image augmented by qualitative numerical results. 6 physical quantities from numerical results of the mathematical model on CCT0: oxygen, necrotic tumor cells, interstitial fluid pressure, radial displacement, the pressure difference between the phases *l* and *t* and tumor cells saturation. Left, at confluence. Right, 85 hours after confluence.

**Fig 8:**
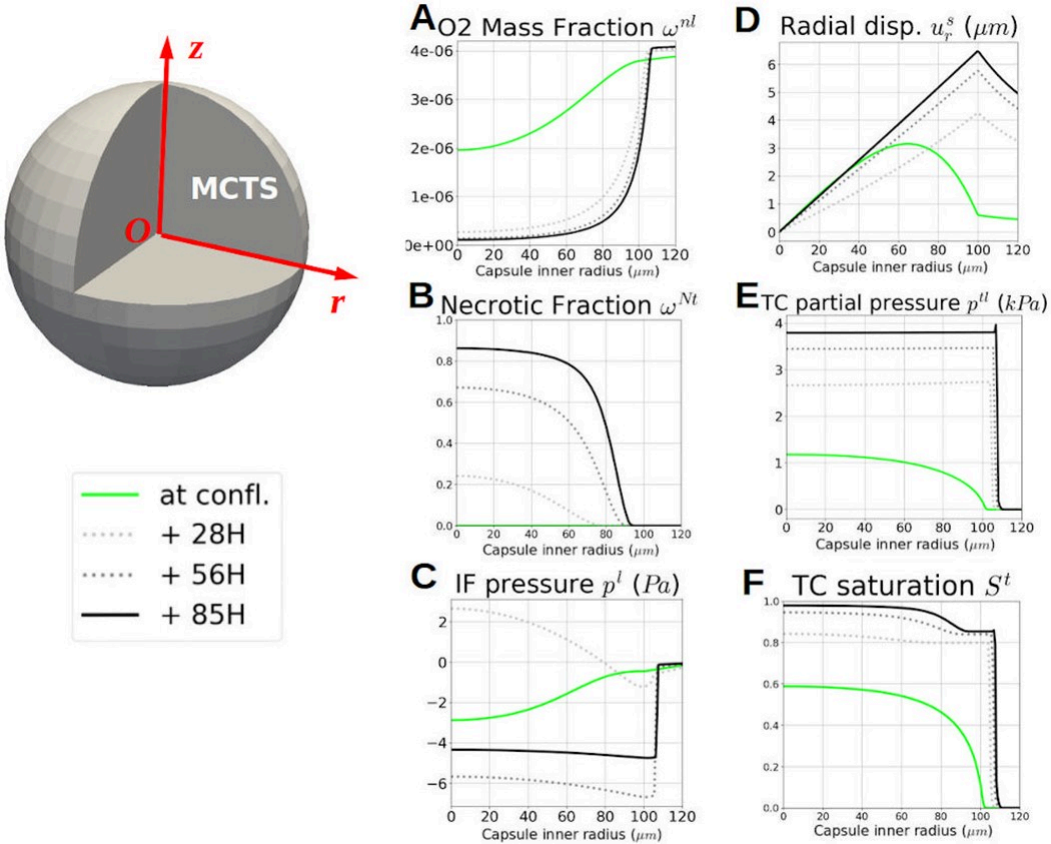
Qualitative numerical results of CCT0 probed along the *r* = *z* line. Quantities probed at confluence, 85 hours after, and two intermediate times (28 and 56 hours): **A** oxygen, **B** necrotic fraction, **C** IF pressure, **D** ECM displacement, **E** TC pressure and **F** TC saturation. **A** and **B**: as mentioned in the experiments, 85 hours after confluence the viable space remaining for TC is a 20 *μ*m thick rim. **C**: after confluence, IF is absorbed by growing TC, provoking a sucking phenomenon, as the cells activity decrease at the MCTS inner core, IF accumulates and its pressure becomes positive. As described in [7], after 2 days of quick growth, the MTCS reaches a state of linear and slow evolution. **D** and **E**: the capsule displacement is driven by TC pressure with the same overall dynamic. **E**, **F** and **B**: relation between the saturation of TC and their necrotic fraction, the TC aggregate density increases with necrotic core.

In addition to overall quantitative results, Figs 7 and 8 provide details on the physical phenomena occurring during growth (from confluence to 85 hours after confluence) of a MTCS encapsulated in a thick capsule with the same geometry as CCT0. These figures quickly allow understanding the importance of physics-based modeling, as they provide qualitative information that could be used to interpret the experimental process as a whole and to better understand the tumor growth process. Fig 7 shows contours of oxygen, necrotic fraction, IF pressure, ECM displacement, TC pressure and TC saturation at confluence and 85 hours later. To gain insight about the dynamics of these quantities, Fig 8 shows them probed along the radius at confluence, 85 hours later, and at two intermediate times (28 and 56 hours).

Fig 8A and 8B show the interplay between oxygen consumption and necrosis. Indeed, as mentioned in the experiments, 85 hours after confluence, the viable space remaining for TC is a 20 *μ*m thick rim. This is explicit in Fig 7, upper right, NT quarter. The comparison of Fig 8B and 8F shows a relation between the saturation of TC and their necrotic fraction. It is a basic experimental fact that, when cells bodies collapse in a necrotic core, the aggregate density increases accordingly. Fig 8D and 8E allow ‘visualizing’ the overall dynamics of the process: the capsule displacement strongly increases after confluence due to the contact with tumor cells which pressure rises from 1.15 kPa at confluence to almost 4 kPa, 85h after confluence. Beyond 85h and until eight days after confluence, it was observed that the tumor cells pressure *p*^*t*^ < *p*_crit_. This is in accordance with the experiment as recorded in [7] where it was reported that the MCTS continued to grow twelve days after confluence, yet at a slow pace.

In the presented physics-based approach, mass conservation is prescribed, so the growing MCTS, which increases in density and size, results in a decrease in interstitial fluid mass. This result, which cannot be measured experimentally, is shown in Fig 8 where a sucking phenomenon due to IF absorption by growing TC can be observed. The Fig 8C shows that after confluence the interstitial fluid pressure becomes positive for a while (see plot relative to 28h). Indeed, after confluence the initial gradient of IF pressure (green line in Fig 8C) reverses since cells in the proliferative peripheral areas move toward the core so IF has to move to the opposite direction, as imposed physically by mass conservation. After 2 days of quick growth, experimentally and numerically, the MTCS reaches a state of linear and slow evolution. From that point onward, the IF flux will not qualitatively change.

**Fig 9:**
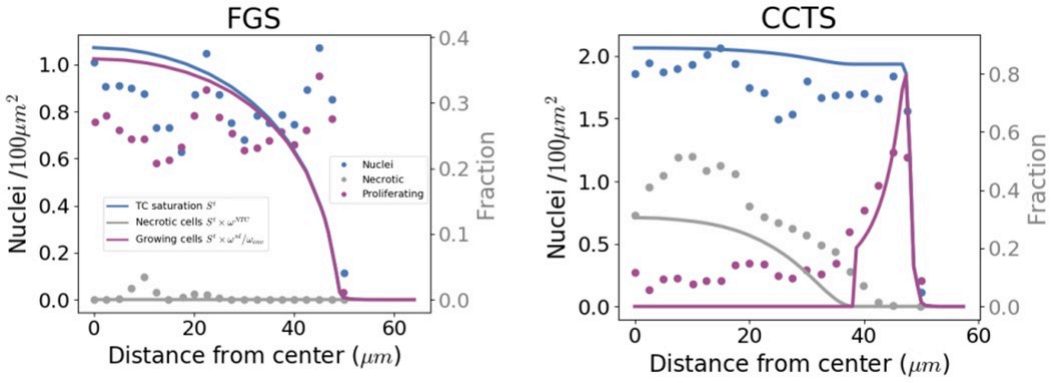
Qualitative comparisons of proliferative and dead cells. Quantification of proliferating and dead cells radial densities for free growth and CCTS: *in vitro-in silico* results. Experimental quantification along the spheroid radius (2.5 *μ* m sampling) of cell nuclei, blue dotted; proliferating cells, purple dotted; dead cells, gray dotted. Numerical quantities: TC Saturation *S*^*t*^, blue plain line; Growing TC fraction $\omega_{\;\text{grow}}^{t}$ (see Eq 32), purple plain line; Necrotic saturation of TC *ω*^*Nt*^ *S*^*t*^, gray plain line. **A**, FGS. **B**, CCTS, **B**, growth (from [7]). TC saturation almost doubles between the two configurations, in encapsulation, necrotic fraction occupy almost half of the TC phase and only a thin rim of the MTCS is viable. CCTS is very far of the conditions of parameters calibration (radius of 50*μ*m *vs*. 100*μ*m), nevertheless *in vitro-in silico* comparison shows a reasonably good agreement, which is quantified Table 2.

To further analyze the reliability of the mathematical model, we also exploit additional data of cell states inside MCTS presented in [7]. More specifically, we reproduced numerically a CT26-MCTS growing in free conditions and the capsule CCTS. Fig 9A and 9B present experimental cell densities (total, proliferative and necrotic, plain lines) at 50 *μ*m radius for the free MCTS and 26h after confluence for CCTS. These experimental results are qualitatively compared with the numerical simulations (Fig 9A and 9B for the free and confined cases respectively, dotted lines). Both configurations show a reasonable agreement with the experimental results, given that none of these quantities have been used for the parameters identification and are very far of the conditions of calibration (radius of 50*μ*m *vs*. 100*μ*m). This is a supplementary argument that showcases the adaptability of this physical-based modeling. The accuracy of the results Fig 9A and 9B have been quantified by RMSE (2.5 *μ*m sampling on experimental data) in Table 2. We used the data available in the free MCTS (FGS) to normalize *S*^*t*^ by the experimental nuclei density at *r* = 0. Unfortunately, in this work, we did not have access to a large sample size to further evaluate our numerical assumptions against experimental data.

**Table 2:** Quantitative comparison between *in vitro-in silico* results, for the control groups FGS and CCTS.

*in vitro-in silico* results	RMSE FGS	RMSE CCTS
nuclei/*S*^*t*^	0.317	0.486
proliferative/$\omega_{\;\text{grow}}^{t}S^{t}$	0.303	0.327
necrotic/*ω*^*Nt*^ *S*^*t*^	0.026	0.238

#### On the experimental estimation of the tumor cell pressure

**Fig 10:**
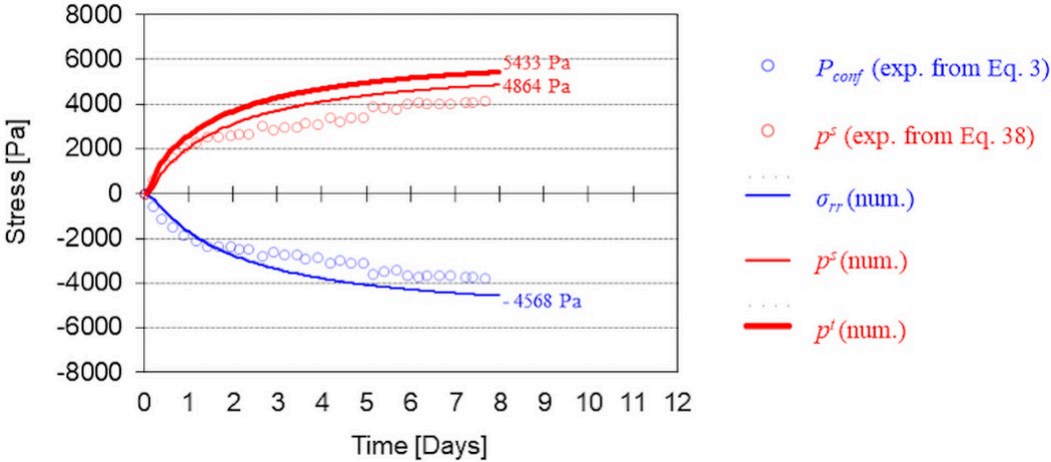
Comparison between numerical stress/pressures and the ones retrieved from the experimental results of CCT0. Blue circles are the confinement pressure obtained experimentally by inverse analysis with Eq 3; red circles is the solid pressure, *p*^*s*^, obtained from the measured displacement using Eq 39; solid lines are *σ*_*rr*_, *p*^*s*^ and *p*^*t*^ obtained numerically.

As explained in the work of Alessandri et al. [7], the pressure in the tumor cell phase is directly related to the confinement pressure arising from the interaction between the capsule and the MCTS calculated with Eq 3. However, from the perspective of porous media mechanics, this confinement pressure essentially corresponds to the total Cauchy stress tensor and not to the pressure in the tumor cell phase. Indeed, the tumor cells pressure *p*^*t*^ does not directly determine the capsule deformation, which is more directly driven by the solid pressure, *p*^*s*^ (see definition Eq 21). The pressure *p*^*s*^ is more representative of the average internal pressure *P*_conf_ obtained experimentally by inverse analysis (see Eq 3). At the confluence time, *p*^*s*^ is significantly lower than the pressure in the tumor cell phase since at that time the MCTS also consists of 40% of IF. After confluence, the saturation of tumor cells *S*^*t*^ increases progressively, so *p*^*s*^ becomes closer to the pressure sustained by the tumor cells. To allow the numerical comparison, we designed a mesh with a subdomain along the internal radius of the capsule to integrate the numerical quantities with FEniCS. Fig 10 shows the comparison between *p*^*s*^, *p*^*t*^ and the *P*_conf_. In the post-confluence stage, it should be noted that the rise of cell pressure induces the deformation of the alginate shell as well as the deformation of the extracellular matrix (constituting the solid scaffold of the MCTS). Thanks to spherical symmetry of the problem, it is easy to derive an equation which allows us calculating the ‘experimental’ solid pressure, *p*^*s*^, from the confinement pressure (calculated with Eq 3) and the stiffness of the ECM (here assumed equal to 1kPa). From the effective stress principle and accounting for assumed elastic constitutive behavior, the solid pressure can be estimated with the following equation

$$\begin{array}{c} p^{s}=P_{\text{conf}}\left(t\right)+3K_{T}\frac{u(R_{\text{in}}\left(t\right))}{R_{\text{in}}\left(t\right)} \end{array}$$ (39)

with *K*_*T*_ bulk modulus of the ECM scaffold.

From the solid pressure, the tumor cell pressure could be estimated by means of an experimental evaluation of the tumor cell saturation degree. The experimental confinement stress evaluated with Eq 3 and the solid pressure evaluated with Eq 39 are depicted in Fig 10 for CCT0. In Fig 10, numerical results for the temporal evolution of *σ*_*rr*_, *p*^*s*^ and *p*^*t*^ within the MCTS (proximity of the alginate shell) are also reported. It can be observed that the final tumor cell pressure is almost 20% higher than the magnitude of the computed radial stress. This difference is however strictly related to the bulk modulus of the ECM scaffold which may also vary during the experiment due to the production of extracellular matrix by tumor cells. The mechanical properties of the ECM have not been investigated in this study. Thus these results are theoretically relevant but only hold a qualitative value.

A deviation of the solid pressure and the radial stress from respective experimentally-derived values can be observed in the figure. The reason is that Eq 3 has been derived assuming a non-compressible alginate while we assumed in our calculation a Poisson ratio *ν* = 0.4. When strain becomes relatively large in the numerical case, the reduction of the thickness due to geometrical non-linearity is smaller than that of the assumed non-compressible case. As a result, the alginate shell is more rigid and the pressure is consequently higher.

## Discussion

The understanding of the tumor cells and mechanical stress interplay is still of critical importance, by its effects on malignant evolution or growth inhibition. The authors of [2] showed that *in vitro* breast tumor cells, under compressive stress between 1 and 5kPa, undergo phenotype switch including apoptosis—cell death—inhibition and structural change of cell shapes, increasing their motility. In [12], by patient MRI analysis, the authors showed that prostate tumor growth is inhibited under compressive stress between 2 and 3kPa. In [7], the growth of the colon tumor cells in the MCTS starts to be inhibited at 2.2kPa and fully inhibited at 4kPa. These results, *in vivo* and *in vitro* on difference cell lines, prove the existence of a window of mechanical stress, where the invasiveness can increase or the growth can stop. Consequently, deeply investigating this range of mechanical constrains could lead to new therapeutic insights.

We showed, in this paper, the *in silico* reproduction of MCTS growth experiments in various physical conditions: free and encapsulated within alginate shells of different sizes and thicknesses. Thanks to a robust validation protocol, variance-based sensitivity analysis, distinct training and validation datasets, all these physical conditions have been successfully simulated by means of a bio-chemo-poromechanical mathematical model. It is important to notice that only one set of parameters, identified on a training dataset (reported in Fig 1B and 1C), has been used for all the numerical simulations performed.

In the frame of the parameter identification process, a local second-order sensitivity analysis has revealed that the parameters of the model almost become independent under confinement (see Fig 6D) within a range of ±10% around the usual literature values. Results of sensitivity analyses also demonstrate that, if the tumor is free to grow, the only influential parameters are the proliferation and the oxygen consumption rates. Conversely, when the tumor growth is constrained by the presence of the alginate capsule, the value of the critical pressure beyond which mechanical stresses inhibit its growth is the main driver. The mathematical model remains reliable even when the growth conditions of the MCTS are modified. This is an advancement with respect of other numerical studies based on poromechanics which are mostly qualitative [23] or solely connected with one reference experimental setup [17].

However, this advancement is only a first step towards a more robust *in vitro*/*in silico* process. On the experimental side, a wider set would raise the confidence on the calibration of the parameters and the validation of the model; repeating this experimental protocol on other standardized cancer cell lines (glioma, breast cancer, prostate cancer, to name a few) would allow enlarging the understanding of this mechano-biological framework and its digital twinning. On the numerical side, if a detailed but local sensitivity analysis accelerates the calibration process, its relevance concerns only a part of the parameter space, and these experimental data exclusively. Attempting global sensitivity without a data bias, or extending these local analyses to other cell lines would be desirable next steps.

The mathematical model is the digital twin MCTS-capsule system since it takes into account its real multiphase nature; hence, the numerical results add new dimensions to the Cellular Capsule Technology. In particular, it is shown that the pressure estimated experimentally illustrates the evolution of the solid pressure, *p*^*s*^, (in the sense of porous media mechanics, see Eq 21) and not of the pressure sustained by the cells, *p*^*t*^. The pressure *p*^*t*^ is always higher than *p*^*s*^ especially during the first phase after confluence (when the MTCS still contains an important volume fraction of IF). It is the direct consequence of the fact that each phase of the MCTS (*i.e*., the ECM, the IF and the TC) has its own stress tensor and that the pressure obtained experimentally by inverse analysis is an average pressure (see Fig 10). The multiphase approach also reveals other behaviors not measurable experimentally. We observe for instance that after confluence occurs a suction of IF from the extra-capsular domain and that cells move from the proliferating rim towards the core of the MCTS where they become necrotic. These results on IF are, for now, only qualitative. The small variation of IF pressure (between −6 and 2Pa, where in living tissue the variation are of the order of 100Pa [32]) requires investigation on *μ*_*l*_, the dynamic viscosity of liquid phase, to quantitatively assess to these variations.

Although these emerging results are inspiring, several physical phenomena are not represented and could lead to valuable insight from the experimental and modeling point of view. The mechanical stress can be the primer of cell necrosis [3] and the CCT experimental framework would be a interesting framework to measure this mechano-biological interplay. The phenotype switch of tumor cells under homeostatic pressure studied in [2] could be modeled and revealed by a alternating CCT growth and free growth on the same aggregate. In this specific study, we hold a line which we wanted simple: i) the number of the modeled physical phenomena is limited; ii) our code is computationally affordable and can be run on an eight-core processor; iii)) our code is adaptable to any geometry and cell lines.

In 2020, mathematical modeling in oncology begins to enter a stage of maturity; today mathematical models of tumor growth tend to clinical applications. Therefore, these must be predictive, funded on measurable or at least quantifiable parameters having a physical meaning. This is what motivated this paper which presents not only a mechanistic bio-chemo-poromechanical model but also a *modus procedendi* to achieve a suitable predicative potential and, with intercession of sensitivity analysis, to quantify relative relevance of mechanisms underlying tumor growth phenomenology.

### Appendices

#### Appendix A. Computational framework

The model has been coded in Python and C++ in the open-source FEniCS framework [31] with an incremental monolithic resolution of the mixed finite element (FE) formulation. The monolithic resolution allows us reducing substantially the computational time compared with staggered resolution methods commonly adopted (*e.g*., see [20]). Whereas spherical symmetry is assumed in experimental results, we have chosen cylindrical symmetry to preserve the generality and the adaptability of the FE mesh and formulation. Even if a 1D spherical formulation would represent an substantial gain in computational efficiency, the resulting mixed problem would not directly be compatible with our FE library.

An updated Lagrangian approach has been adopted to account for geometrical non-linearities, the incremental resolution allows us updating primary variables as follows:

$$\begin{array}{c} X_{n+1}=X_{n}+\delta X \end{array}$$ (40)

with *δ* **X** the vector of unknowns

$$\begin{array}{c} \delta X=(\begin{array}{c} \delta u_{r}^{s}\\ \delta u_{z}^{s}\\ \delta p^{l}\\ \delta p^{tl}\\ \delta \omega^{\bar{nl}} \end{array}) \end{array}$$

After each time step, $X^{s}\in R^{2}$, the geometrical space occupied by the porous medium, is updated as follows:

$$\begin{array}{c} X_{n+1}^{s}=X_{n}^{s}+\delta u^{s} \end{array}$$

### Choice of the element

For all mixed FE problem with vectorial and scalar coupled unknowns, the chosen finite element should verify the inf-sup condition, that is to say, should preserve the coercivity of the bilinear form (see [33] p.223–230). A simple choice is the Taylor-Hood element, with a Lagrange element of order *k* ≥ 1 for the scalar unknowns and order *k* + 1 for the vectorial one. However, modeling an encapsulated tumor growth implies a very sharp gradient at the capsule inner radius for the pressure difference *p*^*tl*^, between *l* and *t* phases. The linear approximation of the Lagrange element of order 1 could not describe it, except at the cost of an extremely refined mesh at the interface, and the error could provoke *numerical infiltration* of tumor cells in the alginate capsule (see S1 Fig). To avoid this phenomenon, the composite Taylor-Hood element has been set to a higher order, more specifically: the mixed FE formulation in FEniCS uses the composite Taylor-Hood element $P_{3}\left(R^{2}\right),{\left[P_{2}\left(R\right)\right]}^{3}$. The demonstration of Lax-Milgram theorem for this type of mixed problem is reported in the *Encyclopedia of Computational Mechanics*, Vol.1, p.149–202 [34].

### Choice of mesh element size

The mixed FE problem has been computed on 5 different meshes, with uniform element sizes *dh* = 50, 20, 10, 5 and 2.5*μ*m. To measure the FE solution degradation the primary variable $\omega^{\bar{nl}}$, the oxygen mass fraction, has been monitored at the spheroid center for 4 days (see S2 Fig). The thinner mesh of element size *dh* = 2.5 *μ*m has been used as a reference for the RMSE. Despite an important increase of the computation time, the mesh element size of *dh* = 5 *μ*m has been chosen to restrict the relative degradation of the FE solution to *RMSE* = 0.01 (see S1 Table).

### Verification of the FE formulation with an analytical solution

If this system is considered with a single-phase flow into a porous medium under a constant load with the right boundary conditions, one obtains the problem denoted Terzaghi’s consolidation, which has an analytical solution [35]. The system, under a constant load **T**, is reduced to two primary variables the displacement of the solid scaffold **u**^*s*^ and the pressure of the single phase fluid *p*^*l*^:

$$\begin{array}{c} \left\{ \begin{array}{c} \nabla .v^{s}-\nabla .[\frac{k}{\mu }\nabla p^{l}]=0\;\text{on}\;\Omega \\ \nabla .\bar{\bar{t_{t}}}=0\;\text{on}\;\Omega \\ \nabla .\bar{\bar{t_{t}}}=-T\;\text{on}\;\Gamma_{s} \end{array}\right. \end{array}$$ (43)

with $T=(\begin{array}{c} 0\\ p_{0} \end{array})$

The fluid is free to escape only at the loaded boundary, this boundary condition is denoted drained condition. The analytical solution of this problem is:

$$\begin{array}{c} \begin{array}{c} p^{l}\left(y,t\right)=p_{0}\frac{4}{\pi }\overset{\infty }{\sum_{k=1}}\frac{{\left(-1\right)}^{k-1}}{2k-1}\text{cos}(\left(2k-1\right)\frac{\pi }{2}\frac{y}{L})\text{exp}({\left(2k-1\right)}^{2}\frac{\pi^{2}}{4}\bar{t}) \end{array} \end{array}$$ (42)

With the characteristic time of the consolidation $\bar{t}$, equal to $\frac{c_{v}t}{L^{2}}$, *L* sets to 100*μ*m and *c*_*v*_, the consolidation coefficient:

$$\begin{array}{c} c_{v}=\frac{k}{\mu_{l}}\left(\lambda +2\mu \right) \end{array}$$

where λ and *μ* are Lamé constants of the solid scaffold, *k* is its intrinsic permeability and *μ*_*l*_ the fluid dynamic viscosity. The sum of the RMSE measured at $\bar{t}=0.01,0.1,0.5,1\;$ (see S4 Fig) with the analytical solution as reference gives ∑*RMSE* = 0.0028. The surface error for different element sizes *dh* and time steps *dt* can be found in S4 Fig(right).

#### Appendix B. Sensitivity analysis

For the sensitivity analysis, the experimental input data were:

- for the free MCTS: the volume monitored over a time span from day 1 to day 4. These data are denoted $Y_{\text{free}}^{\text{exp}}$
- for the encapsulated MCTS CCT0: the capsule radial displacement one day after confluence and the corresponding analytical pressure (i.e. incompressible elastic membrane). We chose the capsule of inner radius = 100*μ*m and thickness = 34*μ*m, presented as the reference case in [7]. These data are denoted $Y_{\text{conf}}^{\text{exp}}$.

We performed a variance-based sensitivity study of the FE solution on the parameters, both on the free and encapsulated MCTS, as follows:

- A first-order analysis, the 7 parameters are disturbed one at a time respectively to an 8-points grid.
- Interaction analysis, the 21 parameters tuples are evaluated at the 2 extreme points of the grid.

All the results were interpreted with a polynomial model in order to quantify their weights in the FE solution variance, referred to as Sobol indices.

#### First-order analysis

Each parameter is disturbed one at a time with respect to the grid [−10, −5, −2, −1, +1, +2, +5, +10]%, giving the corresponding ${\tilde{J}}_{\text{free}}$ and ${\tilde{J}}_{\text{conf}}$. The relative variations of the cost functions were calculated as follows:

$$\begin{array}{c} {\text{Var}}_{\text{free}}=\frac{{\tilde{J}}_{\text{free}}-J0_{\text{free}}}{J0_{\text{free}}}\;\text{and}\;{\text{Var}}_{\text{conf}}=\frac{{\tilde{J}}_{\text{conf}}-J0_{\text{conf}}}{J0_{\text{conf}}} \end{array}$$ (43)

where *J*0_free_ and *J*0_conf_ are the costs with the parameters at their generic values (see S5 Fig). In order to quantify the impact of each parameter, the following linear model was set:

$$\begin{array}{c} \text{Var}=1+\sum_{i}\theta_{i}\alpha_{i} \end{array}$$ (44)

where *α*_*i*_ is an auxiliary parameter ∈[−1, +1] representing the perturbations of the *i*^*th*^ parameter along the grid and *θ*_*i*_ the slope of the variation.

In a first-order analysis, the influence of the *i*^*th*^ parameter is given by the Sobol indices:

$$\begin{array}{c} S_{i}=\frac{\theta_{i}^{2}}{\sum_{i}\theta_{i}^{2}} \end{array}$$ (45)

The results for the free and encapsulated configurations are reported in S2 and S3 Tables.

#### Interaction analysis

As the independence of physical phenomena involved in encapsulated configuration is one our major modeling assessment, the interaction between parameters has also been studied. The 21 tuples have been evaluated at the 2 extreme values of the grid for each configuration. The corresponding polynomial model becomes:

$$\begin{array}{c} \text{Var}=1+\sum_{i}\theta_{i}\alpha_{i}+\sum_{ij,i>j}\theta_{ij}\alpha_{i}\alpha_{j} \end{array}$$ (46)

with the respective Sobol indices:

$$\begin{array}{c} S_{i}=\frac{\theta_{i}^{2}}{\sum_{i}\theta_{i}^{2}+\sum_{ij,i>j}\theta_{ij}^{2}}\;\text{and}\;S_{ij}=\frac{\theta_{ij}^{2}}{\sum_{i}\theta_{i}^{2}+\sum_{ij,i>j}\theta_{ij}^{2}} \end{array}$$ (47)

The results for the free and encapsulated configurations are reported in S4 and S5 Tables.

## Supporting information

S1 FigChoice of the element.Composite Taylor-Hood $P_{2}\left(R^{2}\right),{\left[P_{1}\left(R\right)\right]}^{3}$, green; composite Taylor-Hood $P_{3}\left(R^{2}\right),{\left[P_{2}\left(R\right)\right]}^{3}$, black; interface, gray filled. Along the MCTS radius, the interface shown in gray is the geometrical element which its inner facet is inside the tumor spheroid subdomain and its outer facet in contact with the alginate subdomain. The linear approximation $P_{1}\left(R\right)$ of the pressure difference between *l* and *t* phases at the capsule interface is poor (Left, Day 1) and provoke numerical infiltration of tumor cells into the alginate capsule (Right, Day 3). The quadratic approximation $P_{2}\left(R\right)$ at the capsule interface does not provoke numerical infiltration.(TIF)

S2 FigSensitivity of the solution related to mesh refinement.The numerical oxygen mass fraction, $\omega^{\bar{nl}}$, at the center of the spheroid has been monitored during six days for five different mesh finite element sizes *dh*. (*dh* = 50 *μ*m, black; *dh* = 20 *μ*m, green; *dh* = 10 *μ*m, brown; *dh* = 5 *μ*m, light blue; *dh* = 2.5 *μ*m, purple).(TIF)

S3 Fig Boundary conditions of the Terzaghi’s problem. The load is applied at the top face, where the fluid is free to escape, this is denoted drained condition. The five remaining faces are under slip condition for the displacement **u**^*s*^ and no flux condition for the fluid pressure *p*^*l*^.
(TIF)

S4 FigComparison between analytical solution and numerical result.Left: qualitative comparison between analytical solution of Terzaghi’s problem and FEniCS computation. (4 comparisons at characteristic time of consolidation $\bar{t}=0.01,0.1,0.5,1)$. Right: quantitative comparison, error surface between Terzaghi’s analytical solution and FEniCS computation. (x axis: log_10_ of element size *dh*; y axis: log_10_ of *dt*; z axis: log_10_ of RMSE). The minimum RMSE = 0.0028 is reached at *dh* = 5 *μ*m, *dt* = 1*e*^−4^.(TIF)

S5 FigResults with the 7 parameters at their initial values.

$\gamma_{g}^{t}=4\;.10^{-2},\;$ $\gamma_{0}^{nl}=6\;.10^{-4},\;$ *a* = 800, *p*_1_ = 1800, *p*_crit_ = 4000 (see Table 1). Experimental, green dot; Model, red; Sensitivity evaluation, blue x. Left: FG0; Right: CCT0.(TIF)

S1 Table Relative degradation of the solution due to mesh element size. Measured by root mean square, the reference being the thinner mesh with a mesh element size of *dh* = 2.5 *μ*m.
(PDF)

S2 Table Sobol indices of the first-order sensitivity analysis of the FG0 configuration. (PDF)

S3 Table Sobol indices of the first-order sensitivity analysis of the encapsulated growth configuration CCT0. (PDF)

S4 Table Sobol indices of the interaction sensitivity analysis of the FG0 configuration. (PDF)

S5 Table Sobol indices of the interaction sensitivity analysis of the encapsulated growth configuration CCT0. (PDF)

S1 Data (ZIP)

## References

[1] Solid stress inhibits the growth of multicellular tumor spheroids. Helmlinger G, Netti PA, Lichtenbeld HC, Melder RJ, Jain RK. Nat Biotechnol. 1997;15(8):778–83. doi: 10.1038/nbt0897-778 9255794

[2] Tensional homeostasis and the malignant phenotype. Paszek MJ, Zahir N, Johnson KR, Lakins JN, Rozenberg GI, Gefen A, et al. Cancer Cell. 2005;8(3):241–54. doi: 10.1016/j.ccr.2005.08.010 16169468

[3] Micro-environmental mechanical stress controls tumor spheroid size and morphology by suppressing proliferation and inducing apoptosis in cancer cells. Cheng G, Tse J, Jain RK, Munn LL. PLoS One. 2009;4(2):e4632 doi: 10.1371/journal.pone.0004632 19247489 PMC2645686

[4] The role of mechanical forces in tumor growth and therapy. Jain RK, Martin JD, Stylianopoulos T. Annu Rev Biomed Eng. 2014;16(1):321–46. doi: 10.1146/annurev-bioeng-071813-105259 25014786 PMC4109025

[5] Co-evolution of solid stress and interstitial fluid pressure in tumors during progression: implications for vascular collapse. Stylianopoulos T, Martin JD, Snuderl M, Mpekris F, Jain SR, Jain RK. Cancer Res. 2013 July 1; 73(13): 3833–3841. doi: 10.1158/0008-5472.CAN-12-4521 23633490 PMC3702668

[6] Cells competition in tumor growth poroelasticity. Fraldi M, Carotenuto AR. Journal of the Mechanics and Physics of Solids, 2018; 112, 345–367. doi: 10.1016/j.jmps.2017.12.015

[7] Cellular capsules as a tool for multicellular spheroid production and for investigating the mechanics of tumor progression in vitro. Alessandri K, Sarangi BR, Gurchenkov VV, Sinha B, Kießling TR, Fetler L, et al. Proc Natl Acad Sci U S A. 2013;110(37):14843–8. doi: 10.1073/pnas.1309482110 23980147 PMC3773746

[8] Clinically relevant modeling of tumor growth and treatment response. Yankeelov et al. Science translational medicine, 5, 187–196 (2013) doi: 10.1126/scitranslmed.3005686 23720579 PMC3938952

[9] Introduction to the thermodynamically constrained averaging theory for porous medium systems. Gray WG, Miller CT. 2014th ed. Cham, Switzerland: Springer International Publishing; 2014.

[10] Taking cell-matrix adhesions to the third dimension. Cukierman E, Pankov R, Stevens DR, Yamada KM. Science. 2001;294(5547):1708–12. doi: 10.1126/science.1064829 11721053

[11] Buckling of an epithelium growing under spherical confinement Trushko et al. Developmental Cell, 2020; 54; 655–668 doi: 10.1016/j.devcel.2020.07.019 32800097 PMC7497624

[12] Computer simulations suggest that prostate enlargement due to benign prostatic hyperplasia mechanically impedes prostate cancer growth. Lorenzo G, Hughes TJR, Dominguez-Frojan P, Reali A, Gomez H. Proc Natl Acad Sci U S A. 2019;116(4):1152–61. doi: 10.1073/pnas.1815735116 30617074 PMC6347698

[13] A novel, patient-specific mathematical pathology approach for assessment of surgical volume: application to ductal carcinoma in situ of the breast. Edgerton ME, Chuang Y-L, Macklin P, Yang W, Bearer EL, Cristini V. Anal Cell Pathol (Amst). 2011;34(5):247–63. doi: 10.3233/ACP-2011-0019 21988888 PMC3613121

[14] Transport properties of pancreatic cancer describe gemcitabine delivery and response. Koay EJ, Truty MJ, Cristini V, Thomas RM, Chen R, Chatterjee D, et al. J Clin Invest. 2014;124(4):1525–36. doi: 10.1172/JCI73455 24614108 PMC3973100

[15] Calibrating a predictive model of tumor growth and angiogenesis with quantitative MRI. Hormuth DA, Jarrett AM, Feng X, et al. Ann Biomed Eng 47, 1539–1551 (2019). doi: 10.1007/s10439-019-02262-9 30963385 PMC6544501

[16] Mechanism-based modeling of tumor growth and treatment response constrained by multiparametric imaging data. Hormuth D, Jarrett A, Lima E, McKenna M, Fuentes D, Yankeelov T JCO Clinical Cancer Informatics, 3, 1–10 (2019) doi: 10.1200/CCI.18.00055 30807209 PMC6535803

[17] Predicting the growth of glioblastoma multiforme spheroids using a multiphase porous media model. Mascheroni P, Stigliano C, Carfagna M, Boso DP, Preziosi L, Decuzzi P, et al. Biomech Model Mechanobiol. 2016;15(5):1215–28. doi: 10.1007/s10237-015-0755-0 26746883

[18] Nonlinear modelling of cancer: bridging the gap between cells and tumours. Lowengrub JS, Frieboes HB, Jin F, Chuang Y-L, Li X, Macklin P, et al. Nonlinearity. 2010;23(1):R1–9. doi: 10.1088/0951-7715/23/1/R01 20808719 PMC2929802

[19] A multiphase model for three-dimensional tumor growth. Sciumè G, Shelton S, Gray W, Miller C, Hussain F, Ferrari M, et al. New J Phys. 2013;15(1):015005. doi: 10.1088/1367-2630/15/1/015005 24554920 PMC3926362

[20] A tumor growth model with deformable ECM. Sciumè G, Santagiuliana R, Ferrari M, Decuzzi P, Schrefler BA. Phys Biol. 2014;11(6):065004. doi: 10.1088/1478-3975/11/6/065004 25427284 PMC4632987

[21] Three phase flow dynamics in tumor growth. Sciumè G, Gray WG, Hussain F, Ferrari M, Decuzzi P, Schrefler BA. Comput Mech. 2014;53(3):465–84. doi: 10.1007/s00466-013-0956-2

[22] Saturation–pressure relationships for two- and three-phase flow analogies for soft matter. Sciumè G, Ferrari M, Schrefler BA. Mech Res Commun. 2014;62:132–7. doi: 10.1016/j.mechrescom.2014.10.001

[23] Coupling tumor growth and bio distribution models. Santagiuliana R, Milosevic M, Milicevic B, Sciumè G, Simic V, Ziemys A, et al. Biomed Microdevices. 2019;21(2):33. doi: 10.1007/s10544-019-0368-y 30906958 PMC6686908

[24] Partial pressure of oxygen in the human body: a general review. Ortiz-Prado E, Dunn JF, Vasconez J, Castillo D, Viscor G. Am J Blood Res. 2019;9(1):1–14. 30899601 PMC6420699

[25] Repetitive tissue pO2 measurements by electron paramagnetic resonance oximetry: current status and future potential for experimental and clinical studies. Khan N, Williams BB, Hou H, Li H, Swartz HM. Antioxid Redox Signal. 2007;9(8):1169–82. doi: 10.1089/ars.2007.1635 17536960 PMC2921178

[26] The role of cell lysis and matrix deposition in tumor growth modeling Santagiuliana R., Stigliano C., Mascheroni P., Ferrari M., Decuzzi P., Schrefler B. A. Adv. Model. and Simul. in Eng. Sci. (2015) 2:19 doi: 10.1186/s40323-015-0040-x

[27] Mathematical models of cancer: When to predict novel therapies, and when not to. Brady R, Enderling H. Bull Math Biol. 2019;81(10):3722–31. doi: 10.1007/s11538-019-00640-x 31338741 PMC6764933

[28] Mathematical modelling of microenvironment and growth in EMT6/Ro multicellular tumour spheroids. Casciari JJ, Sotirchos SV, Sutherland RM. Cell Prolif. 1992;25(1):1–22. doi: 10.1111/j.1365-2184.1992.tb01433.x 1540680

[29] Forecasting the growth of multicell tumour spheroids: implications for the dynamic growth of solid tumours. Chignola R, Schenetti A, Andrighetto G, Chiesa E, Foroni R, Sartoris S, et al. Cell Prolif. 2000;33(4):219–29. doi: 10.1046/j.1365-2184.2000.00174.x 11041203 PMC6495301

[30] Classical mathematical models for description and prediction of experimental tumor growth. Benzekry S, Lamont C, Beheshti A, Tracz A, Ebos JML, Hlatky L, et al. PLoS Comput Biol. 2014;10(8):e1003800. doi: 10.1371/journal.pcbi.1003800 25167199 PMC4148196

[31] The FEniCS Project Version 1.5 Alnæs M, Blechta J, Hake J, Johansson A, Kehlet B, Logg A et al. Archive of Numerical Software. 2015;3(100)

[32] Interstitial Fluid Pressure in Normal and Inflamed Pulp. Heyeraas KJ, Berggreen E. Critical Reviews in Oral Biology & Medicine. 1999;10(3):328–336. doi: 10.1177/10454411990100030501 10759412

[33] Mixed finite element methods and applications. Boffi D, Brezzi F, Fortin M. 2013th ed. Berlin, Germany: Springer; 2013.

[34] Encyclopedia of computational mechanics: 6 Volume set, Vol.1. Stein E, de Borst R, Hughes TJR. 2nd ed. Nashville, TN: John Wiley & Sons; 2017.

[35] Theory and Problems of Poroelasticity by Arnold Verruijt [Internet]. Verruijt.net. [cited 2021 Mar 4]. Available from: https://geo.verruijt.net/

[36] Management of an Academic HPC Cluster: The UL Experience Varrette S, Bouvry P, Cartiaux H, Georgatos F. Proc. of the 2014 Intl. Conf. on High Performance Computing & Simulation (HPCS 2014) IEEE; 2014

