# Supplementary figures and images for: Digital twinning of Cellular Capsule Technology: Emerging outcomes from the perspective of porous media mechanics
Source: PLoS One. 2021 Jul 12;16(7):e0254512. doi: 10.1371/journal.pone.0254512 (PMC8274916; doi:10.1371/journal.pone.0254512)

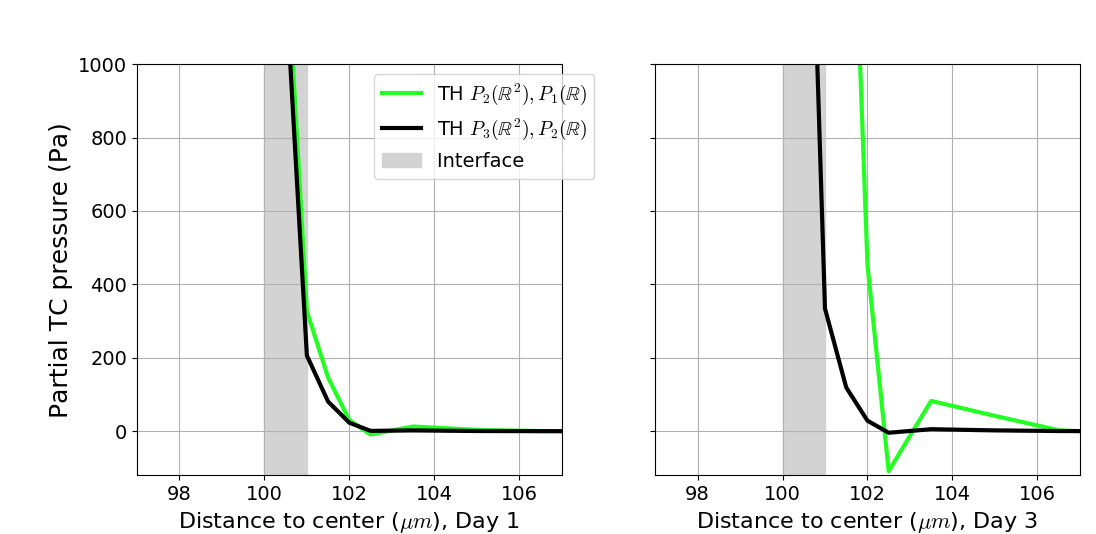

Supplement: S1 Fig — Composite Taylor-Hood P2(R2),[P1(R)]3, green; composite Taylor-Hood P3(R2),[P2(R)]3, black; interface, gray filled. Along the MCTS radius, the interface shown in gray is the geometrical element which its inner facet is inside the tumor spheroid subdomain and its outer facet in contact with the alginate subdomain. The linear approximation P1(R) of the pressure difference between l and t phases at the capsule interface is poor (Left, Day 1) and provoke numerical infiltration of tumor cells into the alginate capsule (Right, Day 3). The quadratic approximation P2(R) at the capsule interface does not provoke numerical infiltration. (TIF) [file pone.0254512.s001.tif]
