# Supplementary material for: Digital twinning of Cellular Capsule Technology: Emerging outcomes from the perspective of porous media mechanics
Source: PLoS One. 2021 Jul 12;16(7):e0254512. doi: 10.1371/journal.pone.0254512 (PMC8274916; doi:10.1371/journal.pone.0254512)
Supplement: S1 Table — Measured by root mean square, the reference being the thinner mesh with a mesh element size of dh = 2.5 μm. (PDF) [file pone.0254512.s006.pdf]

S1 Table. Relative degradation of the solution due to mesh element size. Measured by root mean square, the reference being the thinner mesh with a mesh element size of $dh = 2.5\,\mu$m.

	$dh = 50\,\mu$m	$dh = 20\,\mu$m	$dh = 10\,\mu$m	$dh = 5\,\mu$m
$RMSE(dh, 2.5\,\mu\text{m}, 400/dh)$	0.182	0.032	0.019	0.010
