# Supplementary material for: Digital twinning of Cellular Capsule Technology: Emerging outcomes from the perspective of porous media mechanics
Source: PLoS One. 2021 Jul 12;16(7):e0254512. doi: 10.1371/journal.pone.0254512 (PMC8274916; doi:10.1371/journal.pone.0254512)
Supplement: S2 Table — (PDF) [file pone.0254512.s007.pdf]

S2 **Table.** Sobol indices of the first-order sensitivity analysis of the FG0 configuration.

Parameter	θ	$S_i(\%)$
a	0.2554	6.25
μ_t	-0.1998	3.82
γ_g^t	0.9205	81.17
γ_g^{nl}	-0.1161	1.29
γ_0^{nl}	-0.2790	7.45
p_1	0	0
p_{crit}	0	0
