# Supplementary material for: Digital twinning of Cellular Capsule Technology: Emerging outcomes from the perspective of porous media mechanics
Source: PLoS One. 2021 Jul 12;16(7):e0254512. doi: 10.1371/journal.pone.0254512 (PMC8274916; doi:10.1371/journal.pone.0254512)
Supplement: S3 Table — (PDF) [file pone.0254512.s008.pdf]

S3 Table. Sobol indices of the first-order sensitivity analysis of the encapsulated growth configuration CCT0.

Parameter	θ	$S_i(\%)$
a	0.0550	13.43
μ_t	-0.0006	0.001
γ_g^t	0.0371	6.11
γ_g^{nl}	-0.0056	0.14
γ_0^{nl}	-0.0271	3.27
p_1	0.0279	3.45
p_{crit}	0.1288	73.57
