# Supplementary material for: Digital twinning of Cellular Capsule Technology: Emerging outcomes from the perspective of porous media mechanics
Source: PLoS One. 2021 Jul 12;16(7):e0254512. doi: 10.1371/journal.pone.0254512 (PMC8274916; doi:10.1371/journal.pone.0254512)
Supplement: S4 Table — (PDF) [file pone.0254512.s009.pdf]

S4 Table. Sobol indices of the interaction sensitivity analysis of the FG0 configuration.

Parameter	$S_i(\%)$
a	5.11
μ_t	3.13
γ_g^t	66.42
γ_g^{nl}	1.05
γ_0^{nl}	6.10
p_1	0
p_{crit}	0
Parameter tuples	$S_{ij}(\%)$
$(a,\ \mu_t)$	0.06
$(a,\ \gamma_g^t)$	14.52
$(a,\ \gamma_g^{nl})$	0.02
$(a,\ \gamma_0^{nl})$	1.10^{-4}
$(\mu_t,\ \gamma_g^t)$	1.32
$(\mu_t,\ \gamma_g^{nl})$	0.06
$(\mu_t,\ \gamma_0^{nl})$	0.38
$(\gamma_g^t,\ \gamma_g^{nl})$	1.20
$(\gamma_g^t,\ \gamma_0^{nl})$	0.21
$(\gamma_g^{nl},\ \gamma_0^{nl})$	0.37
