# Supplementary material for: Digital twinning of Cellular Capsule Technology: Emerging outcomes from the perspective of porous media mechanics
Source: PLoS One. 2021 Jul 12;16(7):e0254512. doi: 10.1371/journal.pone.0254512 (PMC8274916; doi:10.1371/journal.pone.0254512)
Supplement: S5 Table — (PDF) [file pone.0254512.s010.pdf]

S5 Table. Sobol indices of the interaction sensitivity analysis of the encapsulated growth configuration CCT0.

Parameter	$S_i(\%)$
a	12.96
μ_t	0.001
γ_g^t	5.89
γ_g^{nl}	0.13
γ_0^{nl}	3.15
p_1	3.33
p_{crit}	70.94
**Parameter tuples**	$S_{ij}(\%)$
$(a,\ \mu_t)$	0.01
$(a,\ \gamma_g^t)$	0.003
$(a,\ \gamma_g^{nl})$	0.01
$(a,\ \gamma_0^{nl})$	0.02
$(a,\ p_1)$	0.009
$(a,\ p_{\text{crit}})$	0.02
$(\mu_t,\ \gamma_g^t)$	0.02
$(\mu_t,\ \gamma_g^{nl})$	5.10^{-6}
$(\mu_t,\ \gamma_0^{nl})$	0.007
$(\mu_t,\ p_1)$	0.003
$(\mu_t,\ p_{\text{crit}})$	0.7
$(\gamma_g^t,\ \gamma_g^{nl})$	0.02
$(\gamma_g^t,\ \gamma_0^{nl})$	0.01
$(\gamma_g^t,\ p_1)$	5.10^{-4}
$(\gamma_g^t,\ p_{\text{crit}})$	0.08
$(\gamma_g^{nl},\ \gamma_0^{nl})$	0.01
$(\gamma_g^{nl},\ p_1)$	0.002
$(\gamma_g^{nl},\ p_{\text{crit}})$	0.87
$(\gamma_0^{nl},\ p_1)$	0.01
$(\gamma_0^{nl},\ p_{\text{crit}})$	1.30
$(p_1,\ p_{\text{crit}})$	0.42
