# Supplementary material for: Digital twinning of Cellular Capsule Technology: Emerging outcomes from the perspective of porous media mechanics
Source: PLoS One. 2021 Jul 12;16(7):e0254512. doi: 10.1371/journal.pone.0254512 (PMC8274916; doi:10.1371/journal.pone.0254512)
Supplement: S1 Data — (ZIP) [file pone.0254512.s011.zip › Data/Source_data_notice.pdf]

Source data notice for the article 'Quantifying the role of mechanics in the free and encapsulated growth of cancer spheroids'

June 19, 2020

1 Data sets for parameters estimation

The two data sets used for the estimation of the parameters, free growth and encapsulated growth in a thick capsule, have been previously published by K Alessandri *et al.*, in "Cellular capsules as a tool for multicellular spheroid production and for investigating the mechanics of tumor progression in vitro". Proc. Natl. Acad. Sci. 110, 14843– 14848 (2013).

2 Data sets for parameters validation

The 4 tests used for the parameters validation have not been published before and are enclosed with this document:

- data_thick_R91_t30.txt
- data_thick_R116_t38.txt
- data_thin_R98_t9.txt
- data_thin_R101_t9.txt

The .txt files contains a three columns array:

Time (Day)   Aggregate radius μm Capsule outer radius μm

Thick capsules are defined as their inner radius is equal to 75% of the outer radius, and thin capsule as their inner radius is equal to 92% of the outer radius. The time when the radius of the aggregate crosses the capsule inner radius is denoted confluency (see Figure 1). The aggregate radius data after confluency are displayed in the main article.

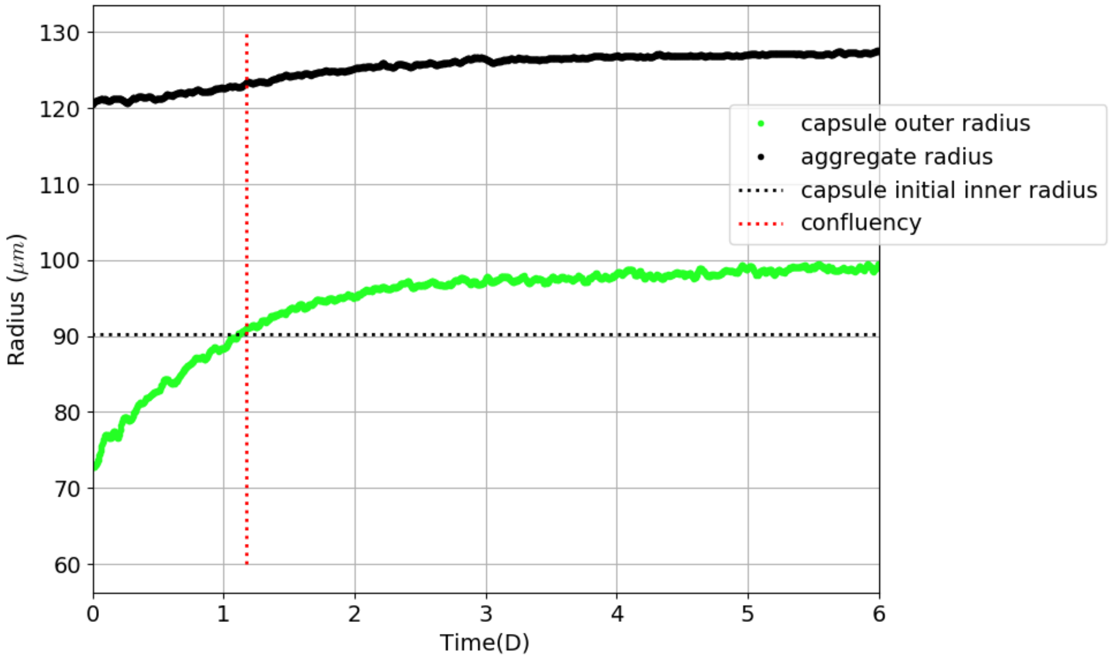

Figure 1: Selection of the post confluency experimental data
